# The C‐terminal tails of GroEL and its mitochondrial and chloroplastic homologs adopt polyproline II helices

**DOI:** 10.1002/pro.70354

**Published:** 2025-10-29

**Authors:** Cristian Segura Rodríguez, Rubén López‐Sánchez, Douglas Vinson Laurents

**Affiliations:** ^1^ Department of Biological Physical Chemistry Institute for Physical Chemistry “Blas Cabrera,” CSIC Madrid Spain

**Keywords:** CD spectroscopy, chaperonins, MD simulations, NMR spectroscopy, polyproline II helix

## Abstract

The chaperonin GroEL and its mitochondrial and chloroplastic homologs mHsp60 and Cpn60 are large barrel‐like oligomeric proteins. Chaperonins facilitate folding by isolating nascent chains in their hollow interior and undergoing ATP‐powered conformational transitions. Due to their vital importance, the structures of GroEL and its homologs were extensively studied by x‐ray crystallography and CryoEM, revealing rings containing seven subunits. Each subunit has three folded domains and a 24 residue C‐terminal extension. Whereas this C‐terminal tail has been reported to bind and stimulate the folding of client proteins, it appears to be blurry or invisible, which suggests disorder. The objective of this study is to characterize conformational preferences in the C‐terminal tails of GroEL, mHsp60 and representative Cpn60s using circular dichroism and nuclear magnetic resonance spectroscopies and molecular dynamics simulations. The tails of GroEL and mHsp60 consist of two segments. The first is rich in residues typical of intrinsically disordered proteins (PKNDAADLGA and PKEEKDPGMG in GroEL and mHsp60, respectively) and the second segment consists exclusively (GroEL) or almost entirely (mHsp60) of Gly and Met residues. The spectroscopic results reveal that these C‐terminal extensions are not wholly disordered but adopt polyproline II helices whose populations are higher in the second Gly/Met‐rich segment. These results are corroborated by MD simulations of GroEL_7_GroES_7_ complexes with ADP or ATP, or ATP and a client protein. Whereas the C‐terminal segments of chloroplastic chaperonins are Gly‐poor, they are rich in proline and also adopt polyproline II helix conformations. These results provide insight into the function of chaperonin C‐terminal tails.

## 1 INTRODUCTION

**FIGURE 1:**
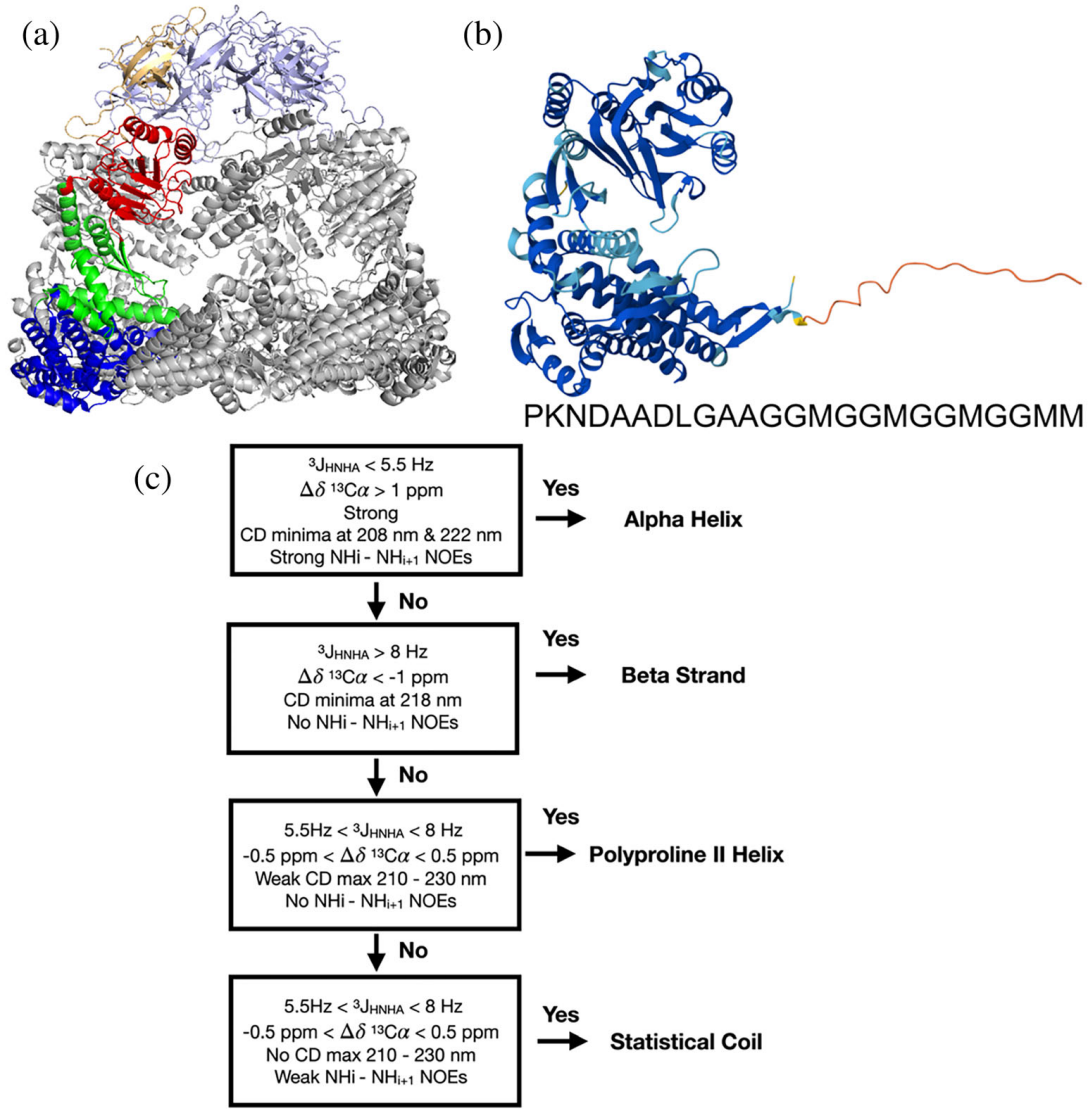
The “disordered” C‐terminal segment in GroEL/mHsp60 and spectral criteria for its conformational characterization. (a) Structure of mHsp60_7_/mHsp10_7_ complex (mitochondrial homolog of GroEL_7_/GroEs_7_): PDB = 6MRD (ref. #3). Six mHsp10 subunits are tinted blue/white; the seventh is colored gold. Six mHsp60 subunits are colored gray; the seventh is colored red, green, and blue for its apical, intermediate, and equatorial domains, respectively. (b) AlphaFold2 structural model (https://alphafold.ebi.ac.uk/entry/P0A6F5) of *Escherichia coli* GroEL (one subunit). Residues whose conformation is predicted with high confidence are colored blue (the three folded domains), and those with low/very low confidence are colored orange and red; these are concentrated in the C‐terminal segment, whose amino acid residue sequence is written. (c) Criteria from NMR data and CD spectral features which can distinguish *α*‐helices, *β*‐strands, PPII helices, and statistical coil ensembles. These criteria are based on those of Ma et al. (2001) but substituting the ^1^H*α* conformational chemical shifts (^1^H*α*Δδ) for ^13^C*α* conformational chemical shifts (^13^CαΔδ), as the latter are more reliable (Wang & Jardetzky, 2002).

Chaperonins are protein folding machines found in all kingdoms of life (Horovitz et al., 2022; Horwich et al., 1990). Group I eubacterial chaperonins, as exemplified by the well‐characterized protein GroEL from *Escherichia coli*, consist of seven subunits, each containing three folded domains. These oligomerize to form a homo‐heptamer with a hollow interior. Unfolded client proteins enter the barrel and bind to its interior surface. The open barrel is then capped by GroES, a smaller homoheptameric protein that acts like a lid. Conformational changes in the complex, which are driven by ATP hydrolysis, GroES binding, and the dimerization of the GroEL_7_·GroES_7_ complex, aid the proper folding of client proteins (Horovitz et al., 2022). GroEL homologs are present in mitochondria (called Hsp60, sequence identity versus GroEL ~60%) with a similar structure (Gomez‐Llorente et al., 2020), and chloroplasts (called Cpn60, sequence identity to GroEL ~60%) (Kim et al., 1994). In addition to the ordered domains, both GroEL and Hsp60 contain C‐terminal tails of about 25 residues in length that appear invisible or diffuse by x‐ray crystallography and CryoEM and are predicted to be disordered by AlphaFold (Figure 1). Whereas the C‐terminal tail was reported to decrease the growth rate but be nonessential in early investigations (McLennan et al., 1993); more recent studies provided convincing evidence from sequence analysis and viability tests performed with modern molecular biology tools that the C‐terminal disordered segment is essential (Kumar et al., 2025). Moreover, these tails contribute to client protein folding (Weaver & Rye, 2014) and form a barrier that blocks clients from escaping through the bottom of the GroEL barrel (Ishino et al., 2015). In GroEL and Hsp60, these tails consist of several charged, short, or polar residues followed by a ~19 residue segment with a remarkably high glycine and methionine content (Supp. Table 1). Recently, a structural class of proteins containing glycine‐rich polyproline II (PPII) helical bundle domains has emerged (Rodríguez & Laurents, 2024). Moreover, the C‐terminal Gly/Met‐rich segments of GroEL and mHsp60 resemble Abductin, an elastic protein present in the hinge ligament of bivalves (Kelly & Rice, 1967), which adopts PPII helix conformations (Bochicchio et al., 2005). This led us to wonder whether the C‐terminal tails of GroEL and mHsp60 might also adopt PPII helical conformations, and here we test this hypothesis.

The chloroplastic chaperonins, which are key for RuBisCO folding (Ellis, 1990), contain two types of subunits, called *α* and *β*, and have been reported to form heptamers (Nishio et al., 1999); which then combine to form heterodecatetramers (Zhao et al., 2019). Like the distinct subunits of cytoplasmic chaperonins (Kim et al., 1994), it is possible that the *α* and *β* subunits of Cpn60 underwent divergent evolution to optimize function (Zhao & Liu, 2017). The chloroplastic subunits also have C‐terminal extensions predicted to be disordered by AlphaFold, but unlike the tails of GroEL and mHsp60, they are glycine‐poor but are often rich in proline residues (Supp. Table 1). Characterizing these segments is a second goal of this study. Group II chaperonins, which are found in Archaea and the cytoplasm of eukaryotes, are more distant cousins of GroEL (Kim et al., 1994). They are generally composed of a dimer of eight or nine subunits. While some group II Archaea chaperonins are homo‐oligomeric, others and eukaryotic group II chaperonins contain slightly different subunits, some of which contain disordered C‐terminal tails rich in glycine, proline, or other charged residues (Supp. Table 1).

## 2 RESULTS

### 2.1 The Gly‐rich stretch of GroEL and mHsp60 contains a high percentage of PPII helix

**FIGURE 2:**
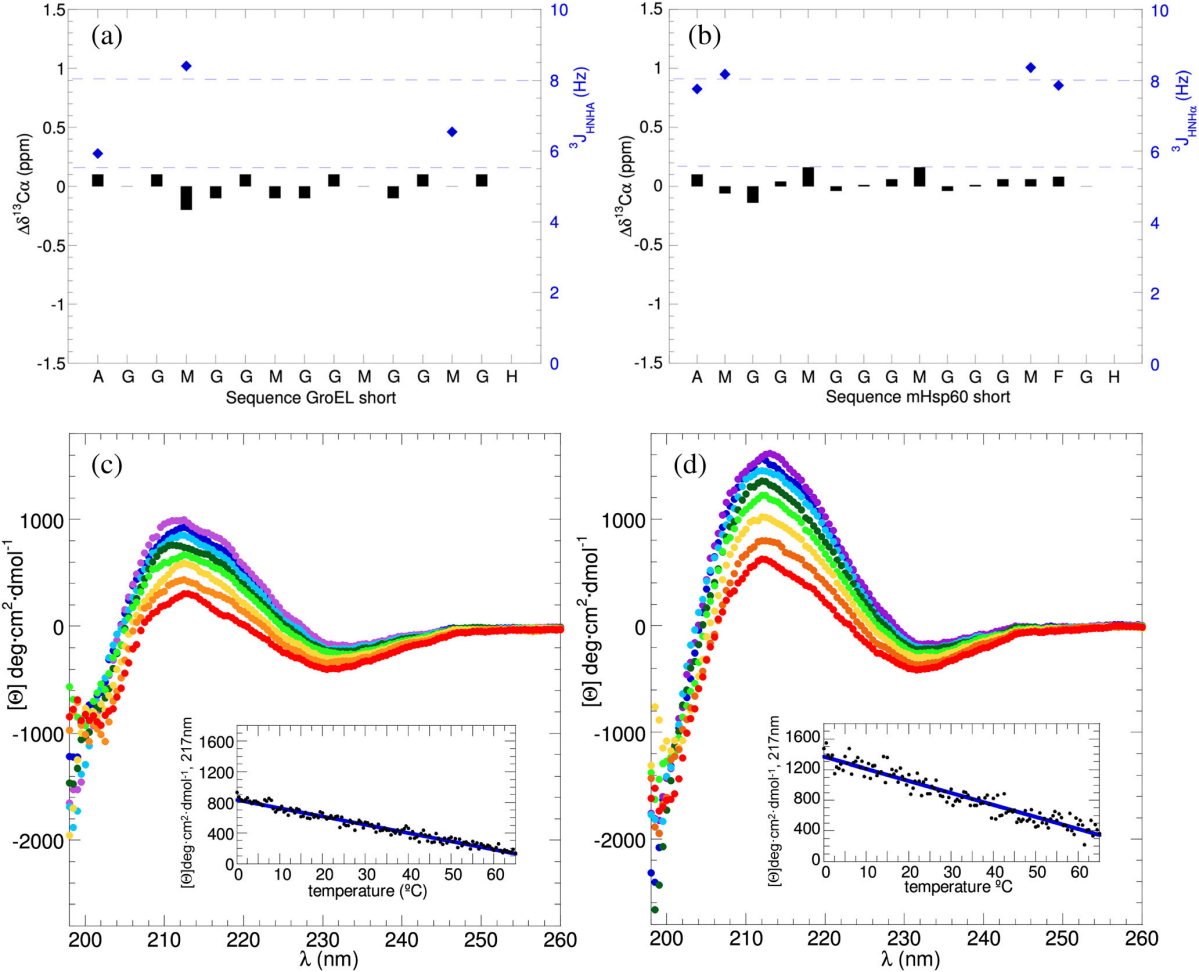
NMR spectral parameters and CD spectra of *GroEL and mHsp60* C‐terminal segments. (a) GroELCtS ^13^C*α* conformation chemical shifts (black bars, left *y*‐axis) and ^3^ *J*_HNHα_ (blue diamonds, right *y*‐axis). As in (b), the latter are measured for non‐overlapping, non‐Glycine residues. (b) mHsp60CtS ^13^C*α* conformation chemical shifts (black bars, left *y*‐axis) and ^3^ *J*_HNHα_ (blue diamonds, right *y*‐axis). (c) Far UV‐CD spectra of GroELCtS recorded at 0°C (violet), 5°C (blue), 10°C (celeste), 17°C (dark green), 25°C (light green), 37°C (yellow), 50°C (orange), 65°C (red). *Inset*: Terminal denaturation of the GroELCtS followed at 217 nm. The fit of a linear equation (blue line) to the data (black points) is [Θ]_217nm_ = 835–10.8 (*T*°C) *R* = 0.98. (d) Far UV‐CD spectra of mHsp60CtS recorded at 0°C (violet), 5°C (blue), 10°C (celeste), 17°C (dark green), 25°C (light green), 37°C (yellow), 50°C (orange), 65°C (red). *Inset*: Heat denaturation of the C‐terminal segment of mHsp60CtS monitored at 217 nm. The fit of a linear equation (blue line) to the data (black points) is [Θ]_217nm_ = 13,200–15.8(*T*°C) *R* = 0.96.

To test for the presence of PPII helix, we studied peptides corresponding to just the Gly/Met‐rich stretch right at the C‐terminus of GroEL and mHsp60. The NMR spectra of these peptides, called GroELCtS and mHsp60CtS, show poor ^1^HN and ^13^Cα chemical shift dispersion and degenerate ^1^Hα glycine signals (Supp. Figure 1). Moreover, the ^13^Cα conformational chemical shifts are very small. These chemical shift data indicate an absence of *α*‐helix and *β*‐strand conformations (Figure 2a,b). The measured ‐Llo ^3^J_HNHα_ couplings are consistent with extended conformations.

The far UV‐CD spectra of GroELCtS and mHsp60CtS, recorded over a range of temperatures from 0 to 65°C, are shown in Figure 2c,d. At low temperatures, the spectra show a maximum near 212 nm which is characteristic of polyamide PPII helices (Gates et al., 2017; Woody, 2009). Upon heating, this maximum weakens in intensity. The CD spectral bands are stronger for mHsp60CtS than GroELCtS. This could be attributed to a greater number of glycine residues in the former, as the intrinsic PPII forming propensity of Gly is higher than Met (Andrews et al., 2020; Kelly et al., 2001). Based on their maximum CD signals of 1000 deg·cm^2^·dmol^−1^ and 1600 deg·cm^2^·dmol^−1^, the populations of PPII helix at 0°C are estimated to be 43% and 47% for GroELCtS and Hsp60CtS, respectively (see Experimental Procedures). At 37°C, the physiological relevant temperature, the %PPII helix would be 41% and 43% for GroELCtS and Hsp60CtS respectively. We then followed the approach of Toal et al. (2014) by recording CD spectra for GroEL CtC and mHsp60 CtC using an augmented bandwidth of 5 nm to access wavelengths down to 185 nm. These spectra confirm the presence of a positive band around 211 nm, which decreases upon heating (Supp. Figure 2). They also reveal a weakening of the negative band around 193 nm at higher temperatures. This is consistent with a loss of polyproline II helical structure as reported by Lopes et al. (2014) who studied these transitions using synchrotron radiation circular dichroism.

Next, the signal at 217 nm was recorded as the sample was heated at 1°C per minute up to 65°C. Instead of the sigmoidal transition typically observed for folded protein, GroELCtS and mHsp60CtS show a linear loss of signal upon heating (Figure 2c,d insets). This is consistent with a non‐cooperative PPII conformation to disordered ensemble transition, which has been previously observed for short peptides (Toal et al., 2014), other isolated PPII helices (Shi et al., 2002) or partly populated PPII helices in intrinsically disordered proteins (Kjaergaard et al., 2010). Furthermore, NMR   ^1^H*α* and ^13^C*α* conformational chemical shifts are very small and similar at 5 and 50°C (Supp. Table 2) and this is consistent with a PPII to coil transition and not a conversion of PPII to α‐helix or *β*‐strand upon heating.

### 2.2 Complete C‐terminal tails contain smaller populations of PPII helix

To further corroborate and assess the position of the PPII helix, we also characterized peptides, called GroELCtC and mHsp60CtC, corresponding to the complete segments of GroEL and mHsp60 that are invisible to x‐ray crystallographic and CryoEM analyses. The assigned NMR spectra of GroELCtC and mHsp60CtC shown respectively in Supp. Figures 3 and 4 reveal a lack of ^1^HN and ^13^C*α* chemical shift dispersion. This is characteristic of an absence of *α*‐helical and *β*‐strand structure (Wang & Jardetzky, 2002; Wishart & Sykes, 1994). Whereas two different ^1^H*α* signals were seen for H‐bonded glycine residues in a folded protein consisting of six PPII helices (Treviño et al., 2018), the two glycine ^1^H*α* signals are degenerate in all GroELCt and mHsp60Ct peptides (Supp. Figures 1, 3, and 4). This is relevant because the seven C‐terminal segments extending from the equatorial domains towards the barrel interior might associate to form a structured bundle of PPII helices. The lack of distinct glycine ^1^H*α* signals suggests they do not. The small ^13^C*α* conformational chemical shifts and ‐L ^3^ *J*_HNHα_ values in the range of 5.5–8.0 Hz (Hagarman et al., 2010; Pardi et al., 1984; Shi et al., 2002) rule out the presence of *α*‐helices or *β*‐strands (Supp. Figure 5). The presence of weak maxima in the CD spectra of GroELCtC and mHsp60CtC (Supp. Figure 5) indicates the presence of PPII helix. These peaks become weaker upon heating, manifesting a partial PPII to coil transition which has been observed previously for other PPII helices (Park et al., 1997; Tiffany & Krimm, 1972). The difference spectra, which were calculated by subtracting spectra recorded at two different temperatures (Supp. Figure 5 insets), are very similar to the CD spectra of peptides (Sreerama & Woody, 1994; Tiffany & Krimm, 1968), collagen (Drzewiecki et al., 2016) and proteins (Gates et al., 2017) known to adopt PPII helices.

Molecular dynamics (MD) simulations corroborate the presence of PPII conformations in the C‐terminal segment of GroEL in the context of GroEL_7_·GroES_7_ complexes. In living bacteria, GroEL is generally found assembled as a barrel‐like heptamer capped by a heptamer of GroES. This double heptamer also dimerizes and binds ATP or ADP and client proteins during the course of its activity. These different states impact the structure of the complex (Skjaerven et al., 2011) and may also affect the conformation of the C‐terminal segments. We therefore performed a series of MD simulations in triplicate with two different force fields (see Materials and Methods) on three different GroEL_7_·GroES_7_ conformational states: (1) seven ADP bound (one ADP per GroEL subunit) with no client proteins; (2) seven ATP bound with no client protein; and (3) seven ATP‐bound with a stable 35‐residue subdomain from the villin protein headpiece domain (McKnight et al., 1996) called “HP35” as a client protein inside the barrel. In the first case, the C‐terminal segments were initially modeled as isolated PPII helices, and in the second and third scenarios, the seven AGGMGGMGGMGGMM C‐terminal segments were assembled into a bilayer of seven PPII helices at the start of the simulations. HP35 is a very small protein subdomain whose folding was thoroughly studied by biophysical methods (Kubelka et al., 2006) and MD simulations, both alone (Lindorff‐Larsen et al., 2011) and inside the GroEL_7_·GroES_7_ complex (Piana & Shaw, 2018). Here, HP35 was included in the third set of simulations to assess the effect of a client protein on the C‐terminal segment's conformation. Finally, to test the effect of bound ADP versus ATP, a fourth set of three 1 μs simulations of GroEL_7_·GroES_7_ with 7 ADP bound and the C‐terminal segments modeled as a seven PPII helical bundle was performed in triplicate with CHARMM36m. We took advantage of a high‐performance computer cluster to tackle these very large, fully hydrated systems, each of which contains almost a half a million atoms. In total, 21 simulations of 1 μs each were run.

**FIGURE 3:**
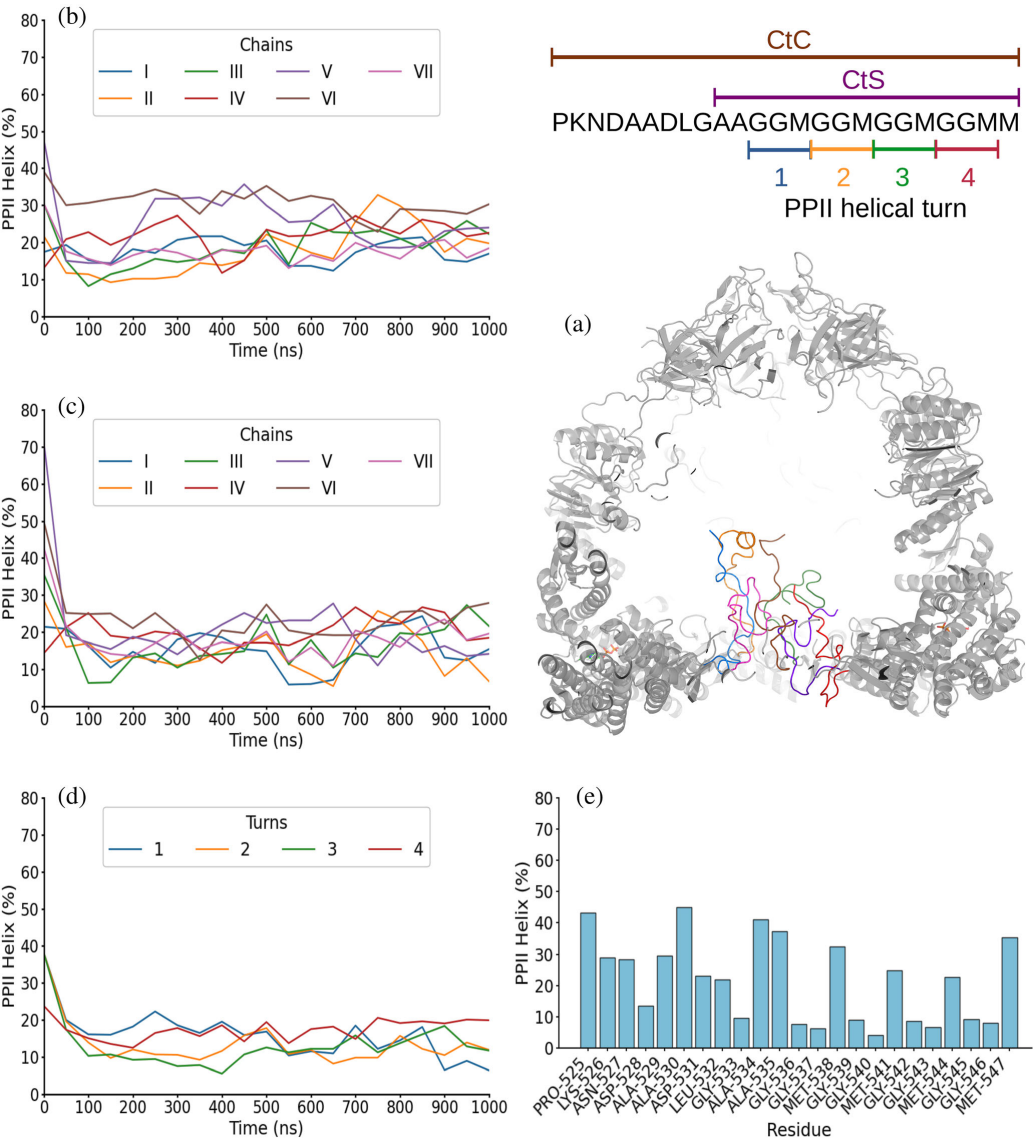
One μs MD simulation of GroEL_7_·GroES_7_·ADP_7_ with Amber99SB‐disp force field. Schematics (top right) show the sequence of the complete (CtC) and short (CtS) C‐terminal sequences and the PPII helix numbering. (a) Frame after 1 μs of simulation showing GroEL_7_·GroES_7_ folded domains in gray, ADP in green, blue, red, and orange for C, N, O, and P atoms, respectively, and the seven C‐terminal segments in blue, orange, green, red, purple, brown, and pink. (b) and (c) PPII helical content averaged over the 50 ns preceding each time point (i.e., data at 50 ns are the average for 0–50 ns) for each of the seven C‐terminal complete (b) or short (c) segments. (d) PPII helical content averaged over the 50 ns preceding each time point in each of the four GGM repeats, colored blue, yellow, green, and red for the first, second, third, and fourth repeats, respectively. (e) Mean per‐residue PPII helical population averaged over the whole 1 μs simulation run. Results from two additional 1 μs simulations are shown in Supp. Figures 6 and 7.

**FIGURE 4:**
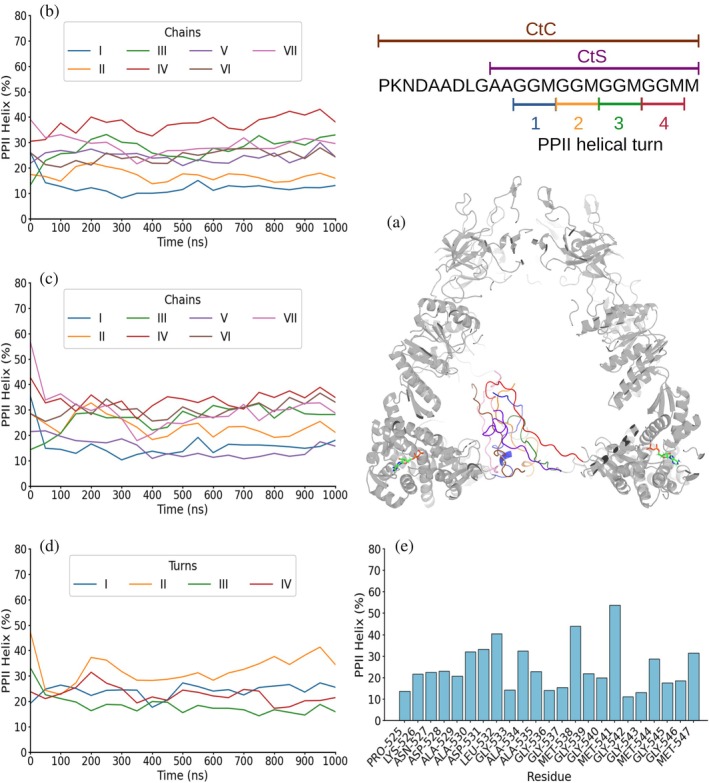
One μs MD simulation of GroEL_7_·GroES_7_·ADP_7_ with CHARMM36m force field. Schematics (top right) show the sequence of the complete (CtC) and short (CtS) C‐terminal sequences and the PPII helix numbering. (a) Frame after 1 μs of simulation showing GroEL_7_·GroES_7_ folded domains in gray, ADP in green, blue, red, and orange for C, N, O, and P atoms, respectively, and the seven C‐terminal segments in blue, orange, green, red, purple, brown, and pink. (b) and (c) PPII helical content averaged over the 50 ns preceding each time point (i.e., data at 50 ns are the average for 0–50 ns) for each of the seven C‐terminal complete (b) or short (c) segments. (d) PPII helical content averaged over the 50 ns preceding each time point in each of the four GGM repeats, colored blue, yellow, green, and red for the first, second, third, and fourth repeats, respectively. (e) Mean per‐residue PPII helical population averaged over the whole 1 μs simulation run. Results from two additional 1 μs simulations are shown in Supp. Figures 8 and 9.

In the first scenario, with ADP bound, the CtC segments of the GroEL_7_·GroES_7_ complex were observed in the Amber99SB‐disp simulations to remain separate and sampled a wide range of conformations, including a population of ~22% PPII helical conformations (Figure 3a,d; Supp. Figures 5A, D and 6A, D). This PPII population decreases to ~16% when analyzing the CtS fragment (Figure 3b; Supp. Figures 6B and 7B). No particular chain or turn (Figure 3c; Supp. Figures 6C and 7C), showed a clear preference to adopt more or less PPII helical conformation. Despite running a total of three 1 μs simulations, no tendency to assemble into a PPII helical bilayer was seen (Supp. Videos 1–3). When the same state was simulated with CHARMM36m, the time‐averaged PPII populations remained similar, ~23% and ~22%, when analyzing the CtC and the CtS fragments of the ADP_7_·GroEL_7_·GroES_7_ complex, respectively (Figure 4a,b,d; Supp. Figures 8A, B, D and 9A, B, D). The seven chains still behaved independently, yet transient two‐ or three‐chain associations appeared but never matured into a persistent bilayer within 1 μs in the three independent simulations (Supp. Videos 4–6). Nevertheless, as with the Amber99SB‐disp force field, none of the GGM motifs that form each of the studied turns showed a greater tendency than the rest to form PPII conformation (Figure 4c; Supp. Figures 8C and 9C).

**FIGURE 5:**
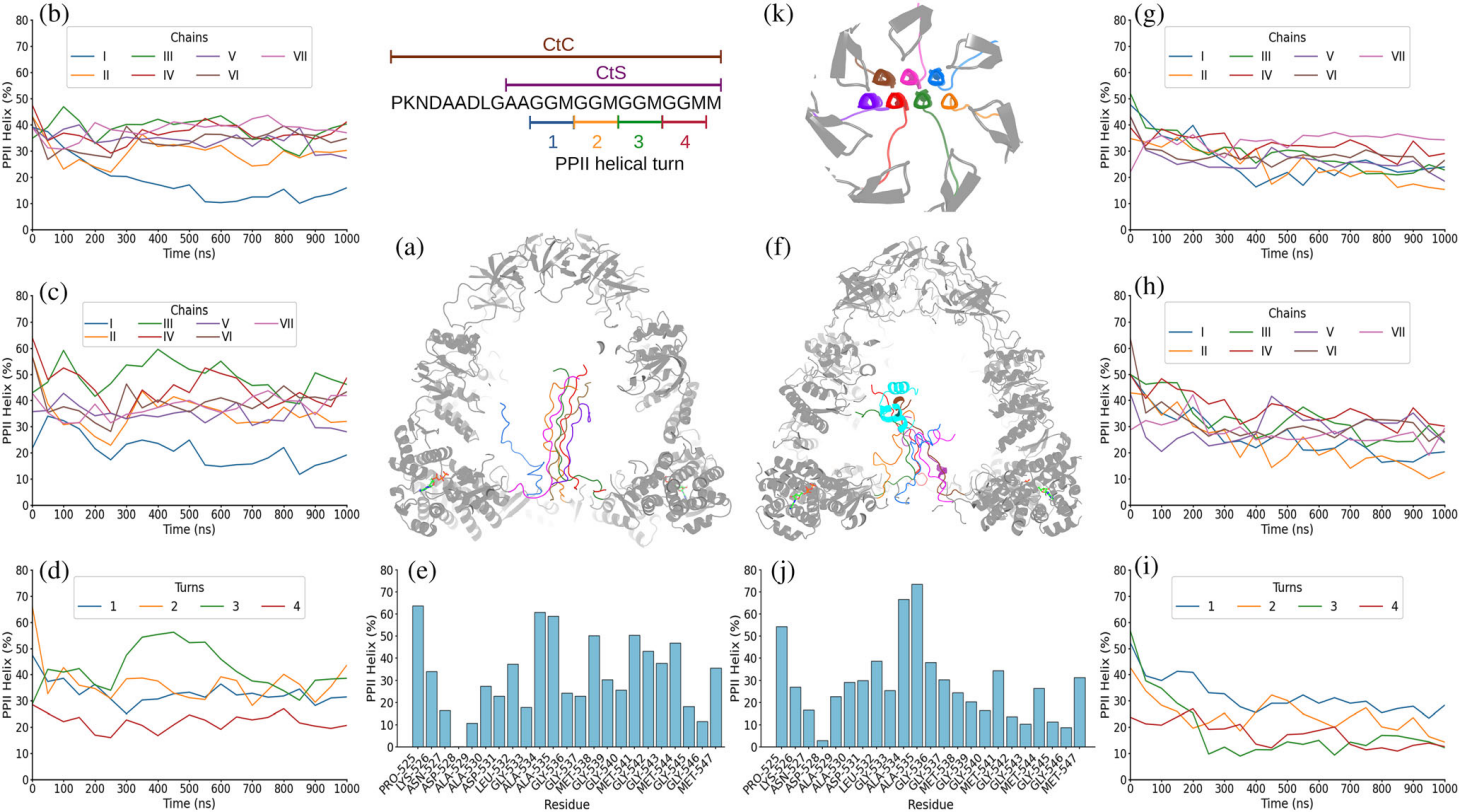
One μs MD simulation of GroEL_7_·GroES_7_·ATP_7_ or GroEL_7_·GroES_7_·ATP_7_ plus HP35 with Amber99SB‐disp force field. Schematic diagrams in the top central part of the figure show the sequence of the complete (CtC) and short (CtS) C‐terminal segments, the numbering of the PPII helical turns, and a top‐down view of the GGM repeats of the seven segments arranged in a PPII helical bundle. GroEL_7_·GroES_7_ with 7 ATP: (a) Frame after 1 μs of simulation of GroEL_7_·GroES_7_ with 7 ATP without HP35 (panels (a)–(d)) showing GroEL_7_·GroES_7_ folded domains in gray, ATP in green, blue, red, and orange for C, N, O, and P atoms, respectively. The C‐terminal segments are colored blue (I), orange (II), green (III), red (IV), purple (V), brown (VI), and pink (VII). (b) and (c) PPII helical content averaged over the 50 ns preceding each time point for each of the seven C‐terminal complete (b) or short (c) segments. (d) PPII helical content averaged over the 50 ns preceding each time point in each of the four GGM repeats, colored blue, yellow, green, and red for the first, second, third, and fourth repeats, respectively. (e) Mean per‐residue PPII helical population averaged over the whole 1 μs simulation run. GroEL_7_·GroES_7_ with 7 ATP + HP35: (f). Frame after 1 μs of simulation with HP35 (in cyan) and GroEL_7_·GroES_7_ folded domains in gray, ATP in green, blue, red, and orange for C, N, O, and P atoms, respectively. The C‐terminal segments are colored blue (I), orange (II), green (III), red (IV), purple (V), brown (VI), and pink (VII). (g) and (h) PPII helical content averaged over 50 ns for each of the seven C‐terminal complete (g) or short (h) segments. (i). PPII helical content averaged over 50 ns in each GGM repeat for the four GGM repeats, colored blue, yellow, green, and red, for the first, second, third, and fourth repeats, respectively. (j) Mean per‐residue PPII helical population averaged over the whole 1 μs simulation run. Results from two additional 1 μs MD simulations are shown in Supp. Figures 10 and 11.

**FIGURE 6:**
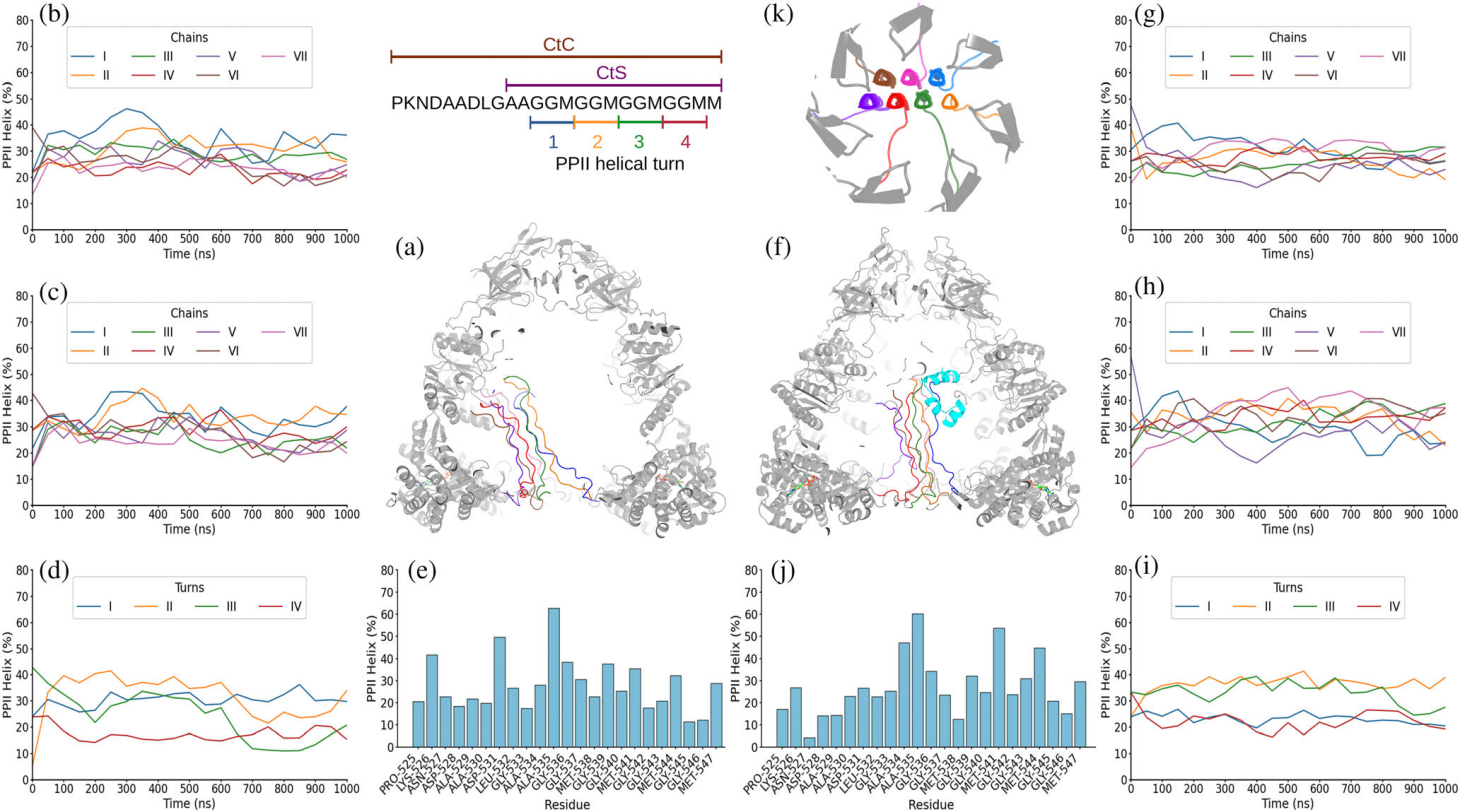
One μs MD simulation of GroEL_7_·GroES_7_·ATP_7_ or GroEL_7_·GroES_7_·ATP_7_ plus HP35 with CHARMM36m force field. Schematic diagrams in the top central part of the figure show the sequence of the complete (CtC) and short (CtS) C‐terminal segments, the numbering of the PPII helical turns, and a top‐down view of the GGM repeats of the seven segments arranged in a PPII helical bundle. GroEL_7_·GroES_7_ with 7 ATP: (a) Frame after 1 μs of simulation of GroEL_7_·GroES_7_ with 7 ATP without HP35 (panels (a)–(d)) showing GroEL_7_·GroES_7_ folded domains in gray, ATP in green, blue, red, and orange for C, N, O, and P atoms, respectively. The C‐terminal segments are colored blue (I), orange (II), green (III), red (IV), purple (V), brown (VI), and pink (VII). (b) and (c) PPII helical content averaged over the 50 ns preceding each time point for each of the seven C‐terminal complete (b) or short (c) segments. (d) PPII helical content averaged over the 50 ns preceding each time point in each of the four GGM repeats, colored blue, yellow, green, and red for the first, second, third, and fourth repeats, respectively. (e) Mean per‐residue PPII helical population averaged over the whole 1 μs simulation run. GroEL_7_·GroES_7_ with 7 ATP + HP35: (f) Frame after 1 μs of simulation with HP35 (in cyan) and GroEL_7_·GroES_7_ folded domains in gray, ATP in green, blue, red, and orange for C, N, O, and P atoms, respectively. The C‐terminal segments are colored blue (I), orange (II), green (III), red (IV), purple (V), brown (VI), and pink (VII). (g) and (h) PPII helical content averaged over 50 ns for each of the seven C‐terminal complete (g) or short (h) segments. (i) PPII helical content averaged over 50 ns in each GGM repeat for the four GGM repeats, colored blue, yellow, green, and red, for the first, second, third, and fourth repeats, respectively. (j) Mean per‐residue PPII helical population averaged over the whole 1 μs simulation run. Results from two additional 1 μs MD simulations are shown in Supp. Figures 12 and 13.

Remarkably, in the second scenario, the assembled bundle of PPII helices with ATP‐bound GroEL_7_·GroES_7_ largely remained together over the three simulations of one μs with both Amber99SB‐disp (Supp. Videos 7–9) and CHARMM36m (Supp. Videos 10–12). In only one out of three simulations of each force field, one helix near the bundle's edge tended to dissociate and explore diverse conformations, while the PPII helical content remained high for the helices located in the bundle interior. This can be reasonably attributed to the edge helices' relatively low number of interhelical contacts. For the Amber99SB‐disp simulations, the time‐averaged PPII helical population of the CtC and CtS segments was ~31% and ~29%, respectively (Figure 5a,b,d; Supp. Figures 10A, B, D, and 11A, B, D). Meanwhile, for the CHARMM36m simulations, the PPII helical conformation increased from ~31% when analyzing the CtC segment of the GroEL_7_·GroES_7_ complex (Figure 6a,d; Supp. Figures 12A, D, and 13A, D) to ~35% for the CtS segment (Figure 6b; Supp. Figures 12B and 13B). These mean PPII helical conformation percentages are similar to that observed experimentally by CD spectroscopy. PPII helix content also varied along the length of the segment; it was usually higher in the first and middle GGM turns (Figures 5c and 6c; Supp. Figures 10C, 11C, 12C, and 13C). The tendency of helix termini to fray could account for this behavior.

In the third scenario, GroEL_7_·GroES_7_ complex binding ATP plus HP35, the PPII helical content of both the CtC and CtS segments appeared to be somewhat lower than when the client protein is not present. This difference is less noticeable for the Amber99SB‐disp simulations, where the PPII helical percentage is respectively reduced to ~29% and ~28% (Figure 5g,h,j; Supp. Figures 10G, H, J and 11G, H, J), than for the CHARMM36m simulations (Figure 6g,h,j; Supp. Figures 12G, H, J and 13G, H, J), where it is reduced to ~28% for the CtC segment and ~30% for the CtS fragment. The lowered PPII helix content was generally most notable for the last GGM repeats (Figures 5j and 6j; Supp. Figures 10J and 11J). Whereas these observations are not consistent with an increased structure formation due to client protein induced crowding effects, they are suggestive of a partial unraveling of the helices to enhance interactions with hydrophobic patches on the client protein. In every independent 1 μs simulation, the client protein was observed to explore a wide variety of positions in the cavity including binding to the C‐terminal tail or the folded subunits of GroEL or GroES with both Amber99SB‐disp (Supp. Videos 13–15) and CHARMM36m (Supp. Videos 16–18).

Finally, three additional 1 μs simulations were performed starting with the seven C‐terminal segments in a pre‐assembled PPII helical bilayer with seven ADP‐bound to GroEL_7_·GroES_7_. Run to compare the effect of the ATP‐ and ADP‐bound states on the PPII helical bundle conformation, these simulations employed the CHARMM36m force field because it gave PPII helical populations in somewhat better agreement to the experimental values than Amber99SB‐disp did. For the preassembled bilayer within the seven ADP‐bound state, Supp. Figures 14–16 show similar PPII helical populations and tendencies as compared to the seven ATP‐bound state (scenario 2). This shows that whether 7 ADP or 7 ATP are bound to the GroEL_7_·GroES_7_ is not important. These results also strongly suggest that whether or not the PPII helical bilayer is pre‐assembled is key.

### 2.3 Chloroplast homologs of GroEL, which are proline‐rich, also adopt PPII conformations

**FIGURE 7:**
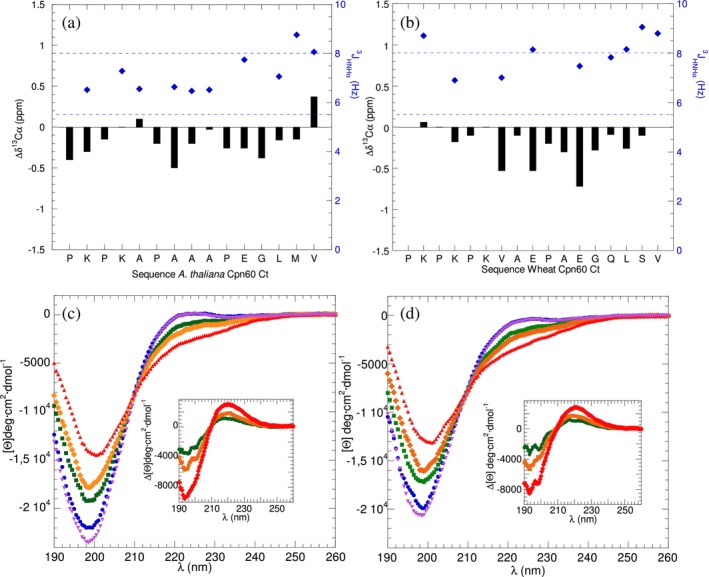
NMR spectral parameters and CD spectra of *Arabidopsis thaliana* and Wheat Cpn60 C‐terminal segments. ^13^C*α* conformational chemical shifts (black bars, left *y*‐axis) and ^3^ *J*_HNHα_ coupling constants (blue diamonds, right *y*‐axis) for the *A. thaliana* (panel a) and wheat (panel b) Cpn60*α* C‐terminal segments. Blue dashed horizontal lines mark the range of constants (between 5.5 and 8.0 Hz) expected for statistical coil or PPII conformations. Far UV‐CD spectra recorded at 5°C (blue), 25°C (green), 37°C (orange), and 65°C (red) and after recooling to 5°C (purple) for the *A. thaliana* (panel c) and wheat (panel d) Cpn60*α* C‐terminal segments. Insets show the difference CD spectra for 5–25°C (green), 5–37°C (orange), and 5–65°C (red).

The NMR spectra of *Arabidopsis thaliana* and wheat Cpn60*α* C‐terminal segments, shown in Supp. Figure 17, reveal a poor ^1^HN chemical shift dispersion, which is consistent with a lack of well‐ordered *α*‐helical or *β*‐sheet structures. There are no detectable ^1^HN_*i*_‐ ^1^HN_*i*+1_ or ^1^HN_*i*_‐ ^1^HN_*i*+3_ NOE signals, which would be characteristic of *α*‐helices. The  ^3^ *J*_HNHα_ coupling constants and small or slightly negative ^13^C*α* conformational chemical shifts are indicative of statistical coil and extended (*β*‐strand or PPII helical) conformations (Figure 7a,b). Similar results are seen for the C‐terminal segments of *A. thaliana* and wheat Cpn60*β* subunits (Supp. Figure 18A). The CD spectra of peptides corresponding to the C‐terminal segments of *A. thaliana* and wheat Cpn60*α* recorded at 5, 25, 37, and 65°C are shown in Figure 7c,d. These spectra show a weak maximum near 224 nm at 5°C, which is suggestive of PPII helical conformations. Except for minor differences in the position of the peak maxima, which could be ascribed to a difference in the content of proline and aromatic residues, the results for the C‐terminal segments of *A. thaliana* and wheat Cpn60β subunits are similar (Supp. Figure 18B, C). Upon heating, the spectra show significant changes, including a decreased signal around 220 nm. Like the difference spectra of GroEL CtC and mHsp60 CtC, the chloroplastic difference spectra closely resemble typical PPII helical spectra. Based on the CD data, at 25°C, the populations of PPII helix in the C‐terminal segments of *A. thaliana* and wheat Cpn60α are about 30% and are slightly higher (32% and 34%) for *A. thaliana* and wheat Cpn60*β*, respectively.

## 3 DISCUSSION

The main finding of this study is that peptides corresponding to the C‐terminal extensions of eubacterial, mitochondrial, and chloroplastic chaperonins, which appear disordered in cryoEM and x‐ray crystallographic analyses, adopt significant populations of PPII conformation in aqueous solution. This is consistent with the high intrinsic propensity of glycine residues to adopt isolated PPII helices (Ohnishi et al., 2006) as well as PPII helical bundles in synthetic peptides (Lotz & Keith, 1971), natural protein domains (De Munck et al., 2021; Dunne et al., 2018; Pentelute et al., 2008) and quasi infinite honeycomb networks (Crick & Rich, 1955).

The CD spectra show that 30%–40% of the structural ensemble adopts a PPII‐like conformation. However, it is unclear whether this is due to single amide groups transiently adopting PPII‐like conformations or to peptides adopting complete PPII helices 30%–40% of the time. Moreover, despite the sequence similarity to the “snow flea” antifreeze protein (Lopes et al., 2014) and abductin (Rodríguez & Laurents, 2024), which contain glycine‐rich PPII helices assembled into bundles, the C‐terminal chaperonin peptides appear to adopt isolated PPII helical conformations under the experimental conditions employed here. Nevertheless, since the seven C‐terminal tails would be present at concentrations of dozens of mM and subject to crowding effects (Rivas & Minton, 2016; Speer et al., 2022) within the chaperonin barrel, the possibility that they may associate under physiological conditions cannot be ruled out and was tested by MD simulations. When each tail was initially modeled as an independent PPII helix in the GroEL·GroES complex, both Amber99SB‐disp and CHARMM36m behaved almost identically: none of the three 1 μs replicas for either force field showed nucleation of a stable bundle PPII helical bundle. However, it is possible that a PPII helical bundle might require more time to assemble. In addition, we have recently shown that hydrogen bond cooperativity, an effect not considered by current MD force fields, substantially stabilizes PPII helical bundles arranged in parallel (López‐Sánchez et al., 2024) or antiparallel (López Sánchez et al., 2025). MD simulations on a pre‐formed bilayer of seven PPII helices showed that this conformation experiences some fraying but is largely stable against complete dissociation and unfolding for up to 1 μs. The pre‐assembled bundle tended to maintain a slightly higher PPII character in the GGM repeats with CHARMM36m than with Amber99SB‐disp, but both force fields behaved similarly for the complete C‐terminal tail, as well as with the isolated PPII helices. In this arrangement, glycine residues are positioned internally and form a network of H‐bonds, and methionine residues are displayed on the surface of the helices where they could interact with client proteins. Previous studies reported the GroEL C‐terminal segment binding (Mamchur et al., 2021) and transient unfolding (Weaver et al., 2017) of client proteins. Adding the client protein HP35 into the MD simulations caused only a modest loss of PPII structure, with similar trends for both force fields and most noticeable at the outer GGM motifs, consistent with partial helix fraying that exposes methionine side chains for substrate interaction. This suggests that partial PPII helix unwinding may increase the ability of methionine side chains to interact with a client protein. Although these interactions could be decreased by the weakened hydrophobic effect present inside the barrel (Korobko et al., 2022), this may be how the C‐terminal segments aid the folding of client proteins. Such an association between PPII helices of the C‐terminal tails could also plug the bottom of the chaperonin barrel and help explain why client proteins do not escape from the barrel (Ishino et al., 2015).

The results from the pre‐assembled PPII helical bilayer in the ADP‐bound state closely mirrored those observed in the ATP‐bound simulations, indicating that the structural context provided by a pre‐assembled bundle plays a more decisive role in stabilizing PPII conformations. Incidentally, the association of the PPII helices formed by Gly‐rich C‐terminal segments of GroEL into putative bundle structures like that advanced here could explain why they are much more resistant to protease K degradation than the C‐terminal segments of chloroplastic Cpn60 (Bai et al., 2015). The latter, being Pro‐rich but Gly‐poor, can form isolated PPII helices but cannot associate (Rodríguez & Laurents, 2024).

The results show significant populations of PPII helix in both the *α* and *β* subunits of the chloroplastic chaperonin Cpn60. Sequence comparison of Cpn60 *α* and *β* subunits (Supp. Table 1) suggests that the C‐terminal extensions of their α subunits often start with three cationic residues and end with two hydrophobic residues. By contrast, *β*‐subunits frequently begin with two anionic residues and end in a SGYGY motif that is reminiscent of the GY segments of TDP‐43 and FUS proteins, which are known to promote *π*/*π* and cation/*π* interactions (Pantoja‐Uceda et al., 2021; Wang et al., 2018). These differences suggest that the C‐terminal tails of the *α* and *β* subunits may have specialized through evolution to fold client proteins rich in anionic and aliphatic (Cpn60*α* subunit) or cationic and aromatic (Cpn60*β* subunit). About 30% of eubacterial genomes contain two or more GroEL homologs (Lund, 2009). In those species, one GroEL retains the Gly/Met‐rich C‐terminal segment and appears to be the essential, principal chaperonin while additional GroEL homologs may contain distinct tail sequences and appear to be important for folding a small number of specific client proteins (Lund, 2009).

These findings reported here for the group I chaperones of eubacteria, chloroplasts, and mitochondria may have some relevance for group II chaperonins found in Archaea and eukaryotic cytoplasm, whose C‐termini frequently contain residues that are invisible to CryoEM and x‐ray crystal diffraction analyses (Cong et al., 2010; Ditzel et al., 1998). These residues are presumed to be disordered and were proposed to form a flexible septum (Ditzel et al., 1998). While distinct among themselves, these C‐terminal segments are often enriched in residues with a high PPII forming propensity (Supp. Table 1).

## 4 MATERIALS AND METHODS

### 4.1 Peptides

Peptides corresponding to the complete GroEL and mHsp60 C‐terminal segments with sequences: acPKNDAADLGAAGGMGGMGGMGGMMKKK and acPKEEKDPGMGAMGGMGGGMGGGMFKKK, respectively, were obtained from CASLO ApS, Denmark. These “complete” C‐terminal peptides are abbreviated as GroELCtC and mHsp60CtC, respectively. These peptides contain three extra Lys residues at the C‐terminus to promote solubility. All peptides were N‐acetylated as indicated by “ac” to mimic the lack of charge in the context of the full‐length protein.

Shorter peptides with just the final Gly/Met‐rich stretch of these peptides were also obtained from Calso ApS. The peptides, named GroELCtS and mHsp60CtS (the last “S” standing for short), have the sequences: acAGGMGGMGGMGGMMH and acAMGGMGGGMGGGMFGH, respectively. For these peptides, one C‐terminal His residue was added to promote solubility.

The N‐acetylated peptides acPKPKAPAAAPEGLMV, acPKPKPKVAEPAEGQLSV, acEPEPVPVGNPMDNSGYGY, and acEPEAAPLANPMDNSGFGY, which correspond to the C‐terminal segments of *A. thaliana* Cpn60*α*, wheat Cpn60*α*, *A. thaliana* Cpn60*β*, and wheat Cpn60*β* subunits, respectively, were purchased from the National Center for Biotechnology (CNB, CSIC), Cantoblanco, Spain.

The sequence and purity of all peptides were verified by mass spectrometry and NMR spectroscopy. Sample concentrations were measured by integration of 1D ^1^H peptide NMR signals to that of a known concentration of sodium trimethylsilylpropanesulfonate, which was also used as the internal chemical shift standard.

A series of NMR spectra; namely: 2D ^1^H‐^1^H COSY, TOCSY, and NOESY and 2D ^1^H‐^13^C HSQC and ^1^H‐^15^N HSQC were recorded on 1–3 mM peptide samples in 10 mM K_2_HPO_4_ buffer (pH 6) and 5°C on the 600 MHz (^1^H) Bruker AvanceNeo spectrometer belonging to the “Manuel Rico” NMR laboratory, IQF/CSIC. The spectral parameters are summarized in Supp. Table 3. The spectra were assigned and analyzed to obtain ^13^C*α* chemical shifts and ^3^ *J*_ΗΝHα_ coupling constants. The ^13^C*α* chemical shifts reveal conformational tendencies (Wishart & Sykes, 1994) when compared to reference ^13^C*α* chemical shifts predicted for a structureless peptide, which were obtained for each peptide and set of experimental conditions using previously reported parameters (Kjaergaard et al., 2011) (Figure 1). ^3^ *J*_HNHα_ couplings also discriminate between *α*‐helical, *β*‐strand, and statistical coil or PPII conformations (Pardi et al., 1984) (Figure 1).

### 4.2 Circular dichroism spectroscopy

Far UV circular dichroism (CD) spectra were recorded on a Jasco 810 spectropolarimeter equipped with a Peltier temperature control unit at 190–260 nm or 198–260 nm with a 50 nm/min scan speed, 1.67 nm bandwidth, peptide concentrations ranging from 130 to 300 μM in 10 mM K_2_HPO_4_ buffer prepared in milliQ water at pH 6. Ten scans were recorded and averaged per spectrum. A reference buffer spectrum was subtracted. Additional far UV CD spectra were recorded from 185 to 260 nm using a 5 nm bandwidth and maintaining the other parameters.

Thermal unfolding experiments were monitored by CD at 217 nm from 0 to 65°C employing a 1°C/min heating rate and a 1.0 nm bandwidth. Spectra were recorded at 0°C before and after the thermal denaturation experiment to test reversibility. The empirical equation of Stellwagen and co‐workers (Park et al., 1997):

(1) $$\%\text{PPII helix}=100\cdot \left(\left[\Theta \right]\max+5560\;\deg\cdot {\mathrm{cm}}^{2}\cdot {\text{dmol}}^{-1}\right)/15140\;\deg\cdot {\mathrm{cm}}^{2}\cdot {\text{dmol}}^{-1}$$

where [Θ]max is the maximum in the spectrum between 210 and 230 nm in deg·cm^2^·dmol^−1^, was used to estimate the population of PPII helix.

### 4.3 Molecular dynamics simulations

Starting models for GroEL_7_·GroES_7_ were based on PDB files: 1PCQ (Chaudhry et al., 2003) and 8BKZ (Torino et al., 2023); the structure 1YRF (Chiu et al., 2005) was used for the HP35 client protein. Pymol was used to construct the C‐terminal Gly‐rich segments as disordered or assembled into a seven PPII helix bilayer. Three types of simulations: (1) with ADP bound, C‐terminal segments initially modeled as isolated PPII helices and no client protein (three 1 μs simulations with different starting velocities); (2) with ATP bound, C‐terminal segments arranged into a seven PPII helix bilayer and no client protein present (three 1 μs simulations with different starting velocities); and (3) with ATP bound, C‐terminal segments arranged into a seven PPII helix bilayer and HP35 present as the client protein (three 1 μs simulations with different starting velocities) were run at 300 K and 1 atm. Each of these three types of experiments was performed with two different force fields for a total of 18 (three types of experiments × three runs × two force fields) 1 μs simulations. The protein force fields AMBER99SB‐disp (Robustelli et al., 2018) and CHARMM36m (Huang et al., 2017), within the CHARMM36 2022 release, were used for their ability to accurately simulate ordered and disordered protein conformations and their interconversions. In the context of Amber99SB‐disp, we applied GAFF2 force field parameters (Wang et al., 2004) and AM1‐BCC atomic charges (Jakalian et al., 2002) for ADP and ATP molecules using ANTECHAMBER (Wang et al., 2006) as implemented in AmberTools23 (Case et al., 2023) and ACPYPE (da Sousa Silva & Vranken, 2012) to enable integration into GROMACS version 2023.1 (Dijkstra et al., 1993). By contrast, no additional work was needed when using CHARMM36, since ADP and ATP are included as standard nucleotide residues (Pavelites et al., 1997; Denning et al., 2011). Finally, three additional 1 μs replicates were run starting with the C‐terminal segments pre‐assembled as a PPII helical bilayer within the ADP‐bound state.

Chaperonin complexes binding seven ADP or ATP molecules with seven Mg^+2^ were placed in a 17 nm cube with Amber99SB‐disp water model (TIP4P‐D with increased C6) or the CHARMM‐modified TIP3P water model, and 150 mM KCl. After steepest descent energy minimization, each system was pre‐equilibrated for 1 ns under NVT and then 1 ns under the NpT ensemble, using a modified Berendsen thermostat (Berendsen et al., 1984) and a Parrinello‐Rahman barostat (Parrinello & Rahman, 1981). Simulations were run in the NpT ensemble, controlling the temperature and pressure using the same algorithms as during the equilibrium phase. The electrostatic interactions were calculated using the Particle Mesh Ewald algorithm, with a 1.2 nm cut‐off radius (Darden et al., 1993). The cut‐off for van der Waals forces was also 1.2 nm. The integration time step was 2 fs as enabled by the LINCS algorithm (Hess et al., 1997).

The simulations were analyzed by extracting and observing frames at each 1 ns using Pymol (DeLano, 2002) and the Supp. Videos were prepared using VMD (Humphrey et al., 1996). In‐house scripts were utilized to measure the percent of PPII helix defined as dihedral angles of −75°± 20° for φ and 150°± 20° for ψ. Structural models of GroEL and mHsp60 to illustrate the disordered nature of the C‐terminal tail (Figure 1b) were generated using AlphaFold2 (Tunyasuvunakool et al., 2021).

## AUTHOR CONTRIBUTIONS

**Cristian Segura Rodríguez:** Investigation; data curation; writing – review and editing. **Rubén López‐Sánchez:** Conceptualization; investigation; writing – review and editing; methodology; validation; visualization; formal analysis; data curation; software; resources; supervision. **Douglas Vinson Laurents:** Conceptualization; investigation; funding acquisition; writing – original draft; methodology; validation; visualization; writing – review and editing; formal analysis; project administration; data curation; supervision; resources.

## FUNDING INFORMATION

This study is part of projects PID2019 109306RB‐I00 and PID2022‐137806OB‐I00, funded by the Spanish Ministry of Science, Innovation and Universities: MICIN/AEI/10.13039/501100011033/FEDER, UE. NMR experiments were performed in the “Manuel Rico” NMR laboratory (LMR) of the Spanish National Research Council (CSIC), a node of the Spanish Large‐Scale National Facility (ICTS R‐LRB). This research project was made possible through the access granted by the Galician Supercomputing Center (CESGA) to its supercomputing infrastructure. The supercomputer FinisTerrae III and its permanent data storage system have been funded by the NextGeneration EU 2021 *Plan de Recuperación, Transformación y Resiliencia*, ICT2021‐006904, and also from the *Programa Operativo Plurirregional de España 2014‐2020* of the European Regional Development Fund (ERDF), ICTS‐2019‐02‐CESGA‐3, and from the *Programa Estatal de Fomento de la Investigación Científica y Técnica de Excelencia del Plan Estatal de Investigación Científica y Técnica y de Innovación* 2013‐2016 (1) *Subprograma estatal de infraestructuras científicas y técnicas y equipamiento* of ERDF, CESG15‐DE‐3114.

## Supporting information


**Data S1:** Supporting Information

**Data S2:** Supporting Information

## Data Availability

The data that supports the findings of this study are available in the supplementary material of this article.
