# Supplementary material for: The C‐terminal tails of GroEL and its mitochondrial and chloroplastic homologs adopt polyproline II helices
Source: Protein Sci. 2025 Oct 29;34(11):e70354. doi: 10.1002/pro.70354 (PMC12571646; doi:10.1002/pro.70354)
Supplement: Supplementary file 1 — Data S1: Supporting Information [file PRO-34-e70354-s001.docx]

**Supplementary Material for**

The C-terminal tails of GroEL and its mitochondrial and chloroplastic homologs adopt polyproline II helices.

Cristian Segura Rodríguez^1^, Rubén López-Sánchez ^1^ & Douglas Vinson Laurents^1*^, *Institute for Physical Chemistry “Blas Cabrera”, CSIC*

*Serrano 119, 28006 Madrid, Spain*

**e-mail for correspondence:*

**Contents**

**I. Supporting Table 1:** Sequences of Representative Chaperonin C-terminal Tails

**II. Supporting Table 2:** Conformational Chemical Shifts of GroELCtS & mHsp60CtS

**III. Supporting Table 3:** NMR spectral parameters.

**IV. Supporting Figure 1:** NMR spectra of GroELCtS and mHsp60CtS

**IV. Supporting Figure 2**: Far UV-CD spectra to 185 nm of GroELCtS and mHsp60CtS

**VI. Supporting Figure 3:** NMR Spectra of GroELCtC

**VII. Supporting Figure 4:** NMR Spectra of mHsp60CtC

**VIII. Supporting Figure 5:** NMR Parameters and CD Spectra of GroELCtC and mHsp60CtC Segments

**IX. Supporting Figure 6:** Second one μs MD simulation of GroEL_7_·GroES_7_·ADP_7_ with Amber99SB-disp force field

**X. Supporting Figure 7:** Third one μs MD simulation of GroEL_7_·GroES_7_·ADP_7_ with Amber99SB-disp force field

**XI. Supporting Figure 8:** Second one μs MD simulation of GroEL_7_·GroES_7_·ADP_7_ with CHARMM36m force field

**XII. Supporting Figure 9:** Third one μs MD simulation of GroEL_7_·GroES_7_·ADP_7_ with CHARMM36mp force field

**XIII. Supporting Figure 10**: Second one μs MD simulation of GroEL_7_·GroES_7_·ATP_7_ or GroEL_7_·GroES_7_·ATP_7_ plus HP35 with Amber99SB-disp force field

**XIV. Supporting Figure 11:** Third one μs MD simulation of GroEL_7_·GroES_7_·ATP_7_ or GroEL_7_·GroES_7_·ATP_7_ plus HP35 with Amber99SB-disp force field

**XV. Supporting Figure 12**: Second one μs MD simulation of GroEL_7_·GroES_7_·ATP_7_ or GroEL_7_·GroES_7_·ATP_7_ plus HP35 with CHARMM36m force field

**XVI. Supporting Figure 13:** Third one μs MD simulation of GroEL_7_·GroES_7_·ATP_7_ or GroEL_7_·GroES_7_·ATP_7_ plus HP35 with CHARMM36m force field

**XVII. Supporting Figure 14:** First one μs MD simulation of GroEL_7_·GroES_7_·ADP_7_ with pre-assembled PPII helical bilayer and CHARMM36m force field

**XVIII. Supporting Figure 15**: Second one μs MD simulation of GroEL_7_·GroES_7_·ADP_7_ with pre-assembled PPII helical bilayer and CHARMM36m force field

**XIX. Supporting Figure 16:** Third one μs MD simulation of GroEL_7_·GroES_7_·ADP_7_ with pre-assembled PPII helical bilayer and CHARMM36m force field

**XX. Supporting Figure 17:** NMR Spectra of *A. thaliana* and wheat Cpn60α

**XXI. Supporting Figure 18**: NMR Parameters and CD Spectra of *A. thaliana* and wheat Cpn60β

**XXII**. Link for Supporting Videos 1-18 of MD simulations.

**Supporting Table 1**

Aminoacid sequences of C-terminal disordered segments in *E. coli* GroEL, human mitochondrial mHsp60 and representative chloroplastic Cpn60 α and β subunits. Glycine, proline, aliphatic and aromatic residues are colored green, blue, bold black and purple, respectively.

| **Group I Chaperonins from Eubacteria, Mitochondria and Chloroplasts** | | |
| --- | --- | --- |
| Chaperonin | Uniprot Code | Sequence |
| *E. coli*  GroEl | P0A6F5 | **P**_525_KNDAAD**LG**AA**GGMGGMGGMGGMGGMM**_548_ |
| *H. sapiens* mitochondrial mHsp60 | P10809 | **P**_550_KEEKD**PGMG**A**MGGMGGGMGGGMF**_573_ |
| *Arabidopsis thaliana*  (Mouse ear cress)  α subunit | P21238 | **P**_571_K**P**KA**P**AAA**P**E**GLMV**_586_ |
| *Triticum aestivum*  (Wheat) Cpn60 α subunit | P08823 | **P**_527_K**P**K**P**KVAE**P**AE**G**Q**L**S**V**_543_ |
| *Fagus sylvatica* (Beech tree) Cpn60 α subunit | A0A2N9GXR7 | K_570_**P**K**P**RS**PV**AA**PP**Q**GL**T**V**_586_ |
| *Citrus sinensis*  (Sweet orange) α | A0A067F287 | K_565_**P**K**P**KTPVAA**PP**Q**GLMV**_582_ |
| *Malus domesticus*  (Apple tree) α | A0A498INK5 | K_632_AK**P**KSAAAAA**P**Q**GM**T**V**_648_ |
| *Eucalyptus grandis*  α subunit | A0A059D558 | K_642_**P**KA**P**AAAS**P**Q**GL**T**V**_656_ |
| *Theobromo cacao*  α subunit, isoform 1 | A0A061GX48 | K_570_**P**K**P**KA**P**AAAAAQA**M**E**I**KDD**P**RK**WL**T**Y**S**G**EARS_602_ |
| *Lactuca sativa*  (Lettuce) α subunit | A0A2J6K5Y6 | K_572_**P**K**P**KA**PM**AAA**P**Q**GM**S**I**_588_ |
| *Oryza sativa*  (Rice) α subunit | Q0INX9 | K_561_**P**K**P**KA**PV**AE**P**AE**G**T**L**T**V**_578_ |
| *Helianthus annuus*  (Sunflower) α subunit | A0A251VPF5 | K_632_**P**K**P**RA**P**RAAE**P**Q**GL**T**V**_648_ |
| Maize α subunit | B7ZZZ2 | K_461_**P**KKA**P**AAAAAAA**P**_474_ |
| *Cicer Arietinum*  (Chickpea) α subunit | A0A1S2YKM3 | K_572_**P**K**P**RA**P**IA**G**S**P**Q**GL**T**V**_588_ |
| *Arabidopsis thaliana*  (Mouse ear cress) β subunit | P21240 | E_583_**P**E**P**V**P**V**G**N**PM**DNS**GYGY**_600_ |
| *Triticum aestivum*  (Wheat) Cpn60 β subunit | A0A3B6I2A6 | **I**_565_KE**P**EAA**PL**AN**PM**DNS**GFGY**_684_ |
| *Fagus sylvatica* (Beech tree) Cpn60 β subunit | A0A2N9F1H9 | K_590_E**P**ESA**VP**A**G**N**PM**DNS**GYGY_609_** |
| *Citrus sinensis*  (Sweet orange) β | A0A067EKN6 | **I**_555_**I**D**P**TK**V**SCC**I**A**IL**_571_ |
| *Malus domesticus*  (Apple tree) β | A0A498K869 | C_589_**VVV**E**I**KE**P**E**P**AV**P**A**G**N**PM**DNS**GYGY**_614_ |
| *Eucalyptus grandis*  β subunit | A0A498K869 | A_591_**I**P**W**TTQ**V**TD**I**E**GF**EDREQ**I**NLD**LL**SEE**V**E_620_ |
| *Theobromo cacao*  β subunit | UPI000848E310 | K_583_E**P**E**P**AAA**G**N**PM**DNS**GYGY**_608_ |
| *Lactuca sativa*  (Lettuce) β subunit | A0A2J6LIA5 | K_585_E**P**E**PMV**A**G**N**PM**DNS**GYGY**_603_ |
| *Oryza sativa*  (Rice) β subunit | A0A0E0HK09 | K_583_E**P**E**P**A**PV**TN**PM**DNS**GYGY**_601_ |
| *Helianthus annuus*  (Sunflower) β subunit | A0A251SG14 | K_587_E**P**E**PV**T**V**GN**PM**DNS**GYGY**_605_ |
| Maize β subunit | A0A3L6DCR4 | K_561_E**P**EAA**PV**AN**PM**DNS**G**KR**H**C**H**_581_ |
| *Cicer Arietinum*  (Chickpea) β subunit | A0A1S3DXB5 | K_575_E**P**E**PVV**A**G**N**PM**DNS**GYGM**_593_ |
| **Group II Chaperonins (**TRiC/CCT**) from Eukaryotic Cytoplasm (*Bos taurus*)** | | |
| bovine TRiC/CCT subunit | Uniprot code | C-terminal sequence  folded & structure known - invisible & presumed to be disordered |
| $\alpha$ | Q32L40 | IDDLIKLHP-ESKDDKHGGYEDAVHSGALD |
| $\beta$ | Q3ZBH0 | NIIKAAPR-KRVPDHHPC |
| $\gamma$ | Q3T0K2 | IVSGHK-KKGDDQSRQGGAPDAGQE |
| $\delta$ | Q2T9X2 | IDDVVNTR-(*nothing*) |
| $\epsilon$ | F1MWD3 | IRKPG-ESEE |
| $\zeta$ | Q3ML7 | DEIMRAG-MSSLKG |
| $\eta$ | Q2NKZ1 | IKNPR-STVDASPAAGRGRGRGRLH |
| $\theta$ | A0A3Q1MTLG | DQIIMAK-LAGGKAPKPGNWDKDGWQDESHI |

**Supporting Table 2:**

**GroEL and mHsp60 CtS Conformational Chemical Shifts at 5ºC and 50ºC.**

**GroELCtS: acAGGMGGMGGMGGMGH***:

|  | Exp $\delta$ (ppm) 5ºC | coil $\delta$ $\dagger$ (ppm) 5ºC | $\Delta\delta$ **(ppm)**  **5ºC** | Exp $\delta$ (ppm) 50ºC | coil $\delta$ $\dagger$ (ppm) 50ºC | $\Delta\delta$ **(ppm)**  **50ºC** |
| --- | --- | --- | --- | --- | --- | --- |
| **Gly ^1^H**$\alpha$ | 3.96 | 4.00 | **-0.04** | 3.96 | 4.00 | **-0.04** |
| **Gly ^13^C**$\alpha$ | 45.22 | 45.37 | **-0.15** | 45.39 | 45.52 | **-0.13** |
| **Ala ^1^H**$\alpha$ | 4.28 | 4.35 | **-0.07** | 4.30 | 4.38 | **-0.08** |
| **Ala ^13^C**$\alpha$ | 52.77 | 52.83 | **-0.06** | 52.63 | 52.73 | **-0.10** |
| **Met ^1^H**$\alpha$ | 4.53 | 4.52 | **+0.01** | 4.50 | 4.50 | **0.00** |
| **Met ^13^C**$\alpha$ | 55.46 | 55.72 | **-0.26** | 55.61 | 55.90 | **-0.29** |

* As the last residue, the chemical shifts of this His are not analyzed.

$\dagger$ The coil chemical shift values were calculated using the approach of Poulsen and coworkers Kjaergaard *et al.* (2010) Prot. Sci. **19**(8) 1555-1564. as implemented on the webserver: https://spin.niddk.nih.gov/bax-apps/nmrserver/Poulsen_rc_CS/ and corrected to 5ºC or 50ºC.

**mHsp60CtS: acAMGGMGGGMGGGMFGH***

|  | Exp $\delta$ (ppm) 5ºC | coil $\delta$ $\dagger$(ppm) 5ºC | $\Delta\delta$ **(ppm)**  **5ºC** | Exp $\delta$ (ppm) 50ºC | coil $\delta$ $\dagger$(ppm) 50ºC | $\Delta\delta$ **(ppm)**  **50ºC** |
| --- | --- | --- | --- | --- | --- | --- |
| **Gly ^1^H**$\alpha$ | 3.97 | 4.00 | **-0.03** | 3.97 | 4.00 | **-0.03** |
| **Gly ^13^C**$\alpha$ | 45.21 | 45.37 | **-0.16** | 45.37 | 45.52 | **-0.15** |
| **Ala ^1^H**$\alpha$ | 4.26 | 4.33 | **-0.07** | 4.28 | 4.36 | **-0.08** |
| **Ala ^13^C**$\alpha$ | 52.61 | 52.60 | **+0.01** | 52.56 | 52.50 | **+0.06** |
| **Met ^1^H**$\alpha$ | 4.53 | 4.52 | **+0.01** | 4.50 | 4.50 | **0.00** |
| **Met ^13^C**$\alpha$ | 55.42 | 55.72 | **-0.30** | 55.62 | 55.89 | **-0.27** |
| **Met ^1^H**$\alpha$ | 4.40 | 4.46 | **-0.06** | 4.38 | 4.43 | **-0.07** |
| **Met ^13^C**$\alpha$ | 55.37 | 55.55 | **-0.18** | 55.60 | 55.74 | **-0.14** |
| **Phe ^1^H**$\alpha$ | 4.62 | 4.67 | **-0.05** | 4.64 | 4.69 | **-0.05** |
| **Phe ^13^C**$\alpha$ | 57.91 | 57.92 | **-0.01** | 57.64 | 57.71 | **-0.07** |

* As the last residue, the chemical shifts of this His are not analyzed.

$\dagger$ The coil chemical shift values were calculated using the approach of Poulsen and coworkers Kjaergaard *et al.* (2010) Prot. Sci. **19**(8) 1555-1564. as implemented on the webserver: https://spin.niddk.nih.gov/bax-apps/nmrserver/Poulsen_rc_CS/ and corrected to 5ºC or 50ºC.

**Supporting Table 3:** NMR Spectral Parameters

| Type of Spectrum | Number of Scans | Sweep Width (ppm) | Matrix Size | Mixing Time (ms) |
| --- | --- | --- | --- | --- |
| 1D ^1^H | 64 | 12 | - | - |
| 2D ^1^H, ^1^H COSY | 16 | 11 x 11 | 8192 x 336 | - |
| 2D ^1^H, ^1^H TOCSY | 16 | 11 x 11 | 2048 x 256 | 40, 60 or 100* |
| 2D ^1^H, ^1^H NOESY | 40 | 11 x 11 | 2048 x 288 | 100-120 |
| 2D ^1^H-^13^C HSQC | 144 | 10.4 x 50 (center 40 ppm) | 1024 x 80 | - |
| 2D ^1^H-^15^N HSQC† | 336 | 10 x 23 | 2048 x 128 |  |

* The default TOCSY mixing time was 60 ms, but some additional experiments were run using shorter or longer mixing times to distinguish and assign longer side chains.

† Recorded only for the longer peptides, namely: GroELCtC and mHsp60CtC.

**Supporting Figure 1**:

NMR spectra of GroELCtS and mHsp60CtS


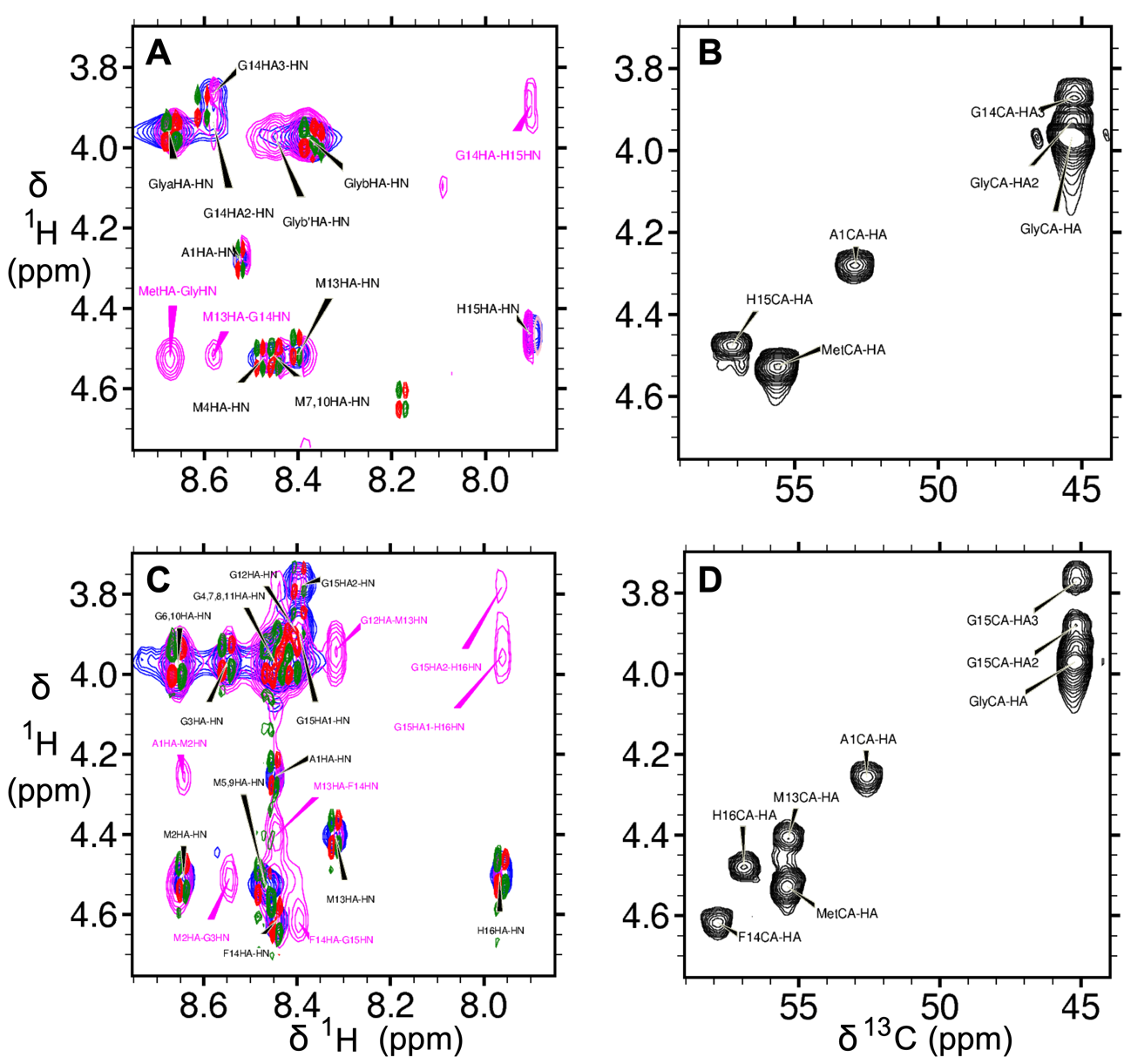


**A**. GroELCtS 2D ^1^H-^1^H COSY (green+/red-), TOCSY (blue) and NOESY (magenta) NMR signals of the HN/Hα region are shown. Intra-residual crosspeaks are labeled in black and inter-residual signals are labeled in magenta.

**B**. 2D ^1^H-^13^C HSQC signals from the ^13^Cα/^1^Hα region of GroELCtS. Overlapped glycine signals are labeled “Gly” and overlapped methionine resonances are labeled “Met”.

**C**. mHsp60 CtC 2D ^1^H-^1^H COSY (green+/red-), TOCSY (blue) and NOESY (magenta) signals of the HN/Hα region are shown. Intra-residual crosspeaks are labeled in black and inter-residual signals are labeled in magenta.

**D**. mHsp60 CtC 2D ^1^H-^13^C HSQC signals from the ^13^Cα/^1^Hα region. Overlapped glycine and methionine signals are labeled “Gly” and “Met” respectively.

**Supporting Figure 2**: Far UV-CD spectra to 185 nm of GroELCtS and mHsp60CtS

**
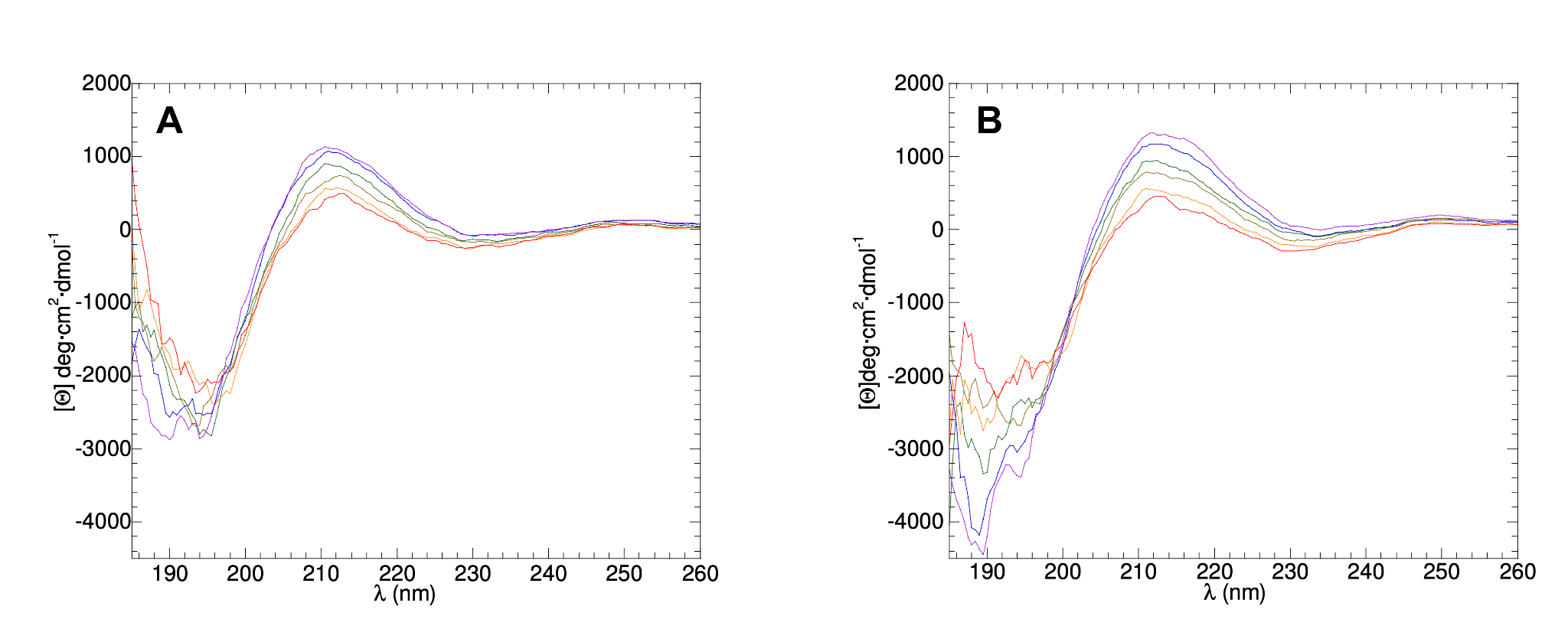
**

**A**. (*left*) Far UV-CD spectra of GroEL CtS recorded with a five nm bandwidth and ten scans at 50 ns/min at 5ºC (blue), 25ºC (green), 37ºC (olive green), 50ºC (orange) 65ºC (red) and, following recooling to -2ºC (purple).

**B**. (*left*) Far UV-CD spectra of mHsp60 CtS recorded with a five nm bandwidth and ten scans at 50 ns/min at 5ºC (blue), 25ºC (green), 37ºC (olive green), 50ºC (orange) 65ºC (red) and, following recooling to -2ºC (purple).

**Supporting Figure 3**: NMR spectra of the long GroELCtC Segment


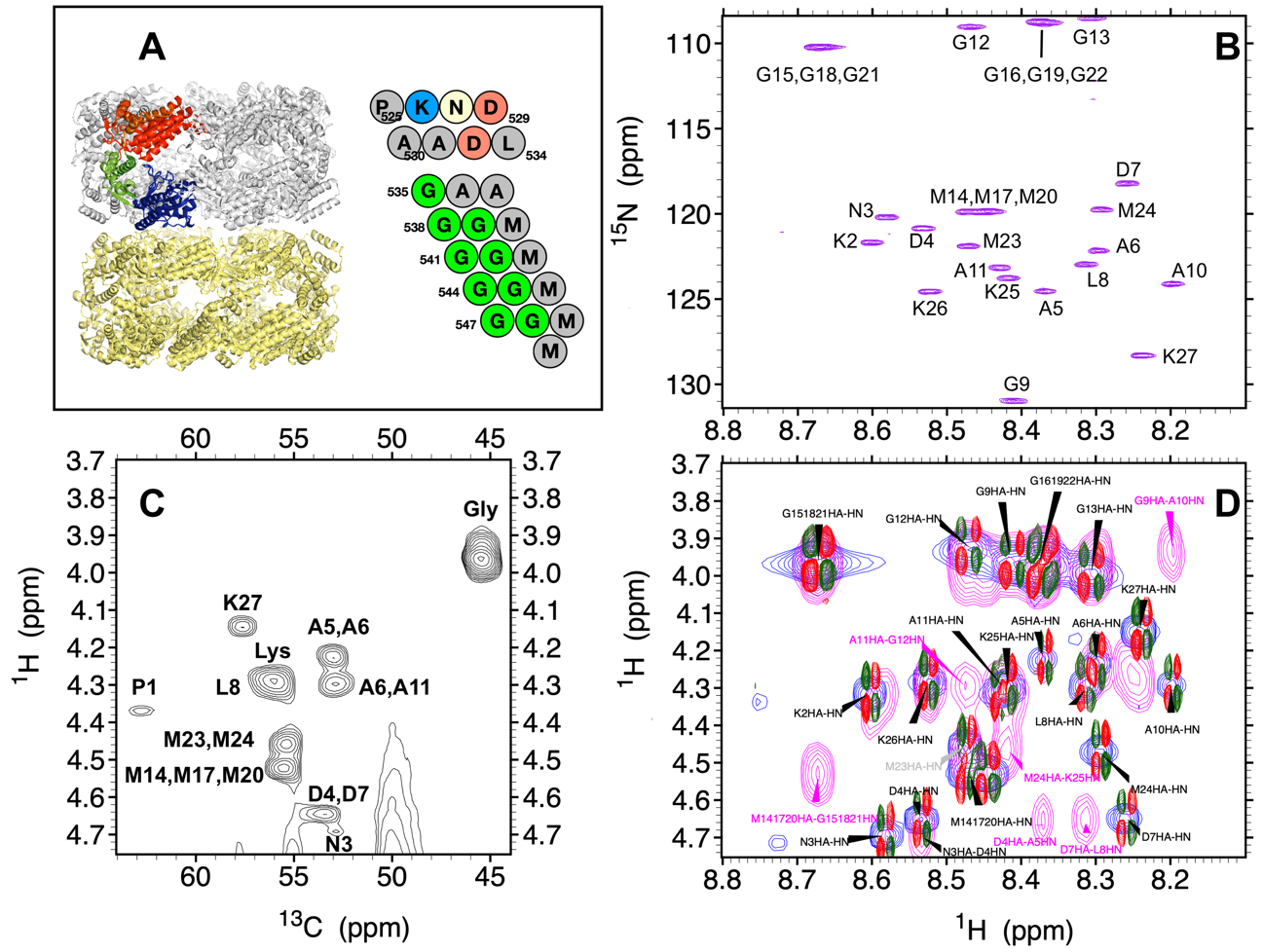


**A**. (*left*) Structure of GroEL (PDB 2EU1) showing seven subunits of the lower barrel in gold and six subunits of the upper barrel in silver. The apical, intermediate and equatorial domains of one subunit are shown in red, green and blue, respectively. (*right*) The residues of the GroEL C-terminal tail, which are invisible to CryoEM or X-ray diffraction.

**B**. ^1^H-^15^N HSQC spectrum of the C-terminal tail of GroEL. The signal of G9 is folded. **C**. ^1^Hα-^13^Cα region of 2D ^1^H-^13^C HSQC spectrum. Peaks with overlapped signals from multiple residues are labeled with the three-letter code (*e.g.* Gly, Lys)

**D**. ^1^HN-^1^Hα region of 2D ^1^H-^1^H COSY (maroon/dark green), TOCSY (blue) and NOESY (magenta) spectra. Inter-residue crosspeaks are colored magenta and intra-residues crosspeaks are colored black.

**Supporting Figure 4**: NMR spectra of mHsp60 C-terminal Segment


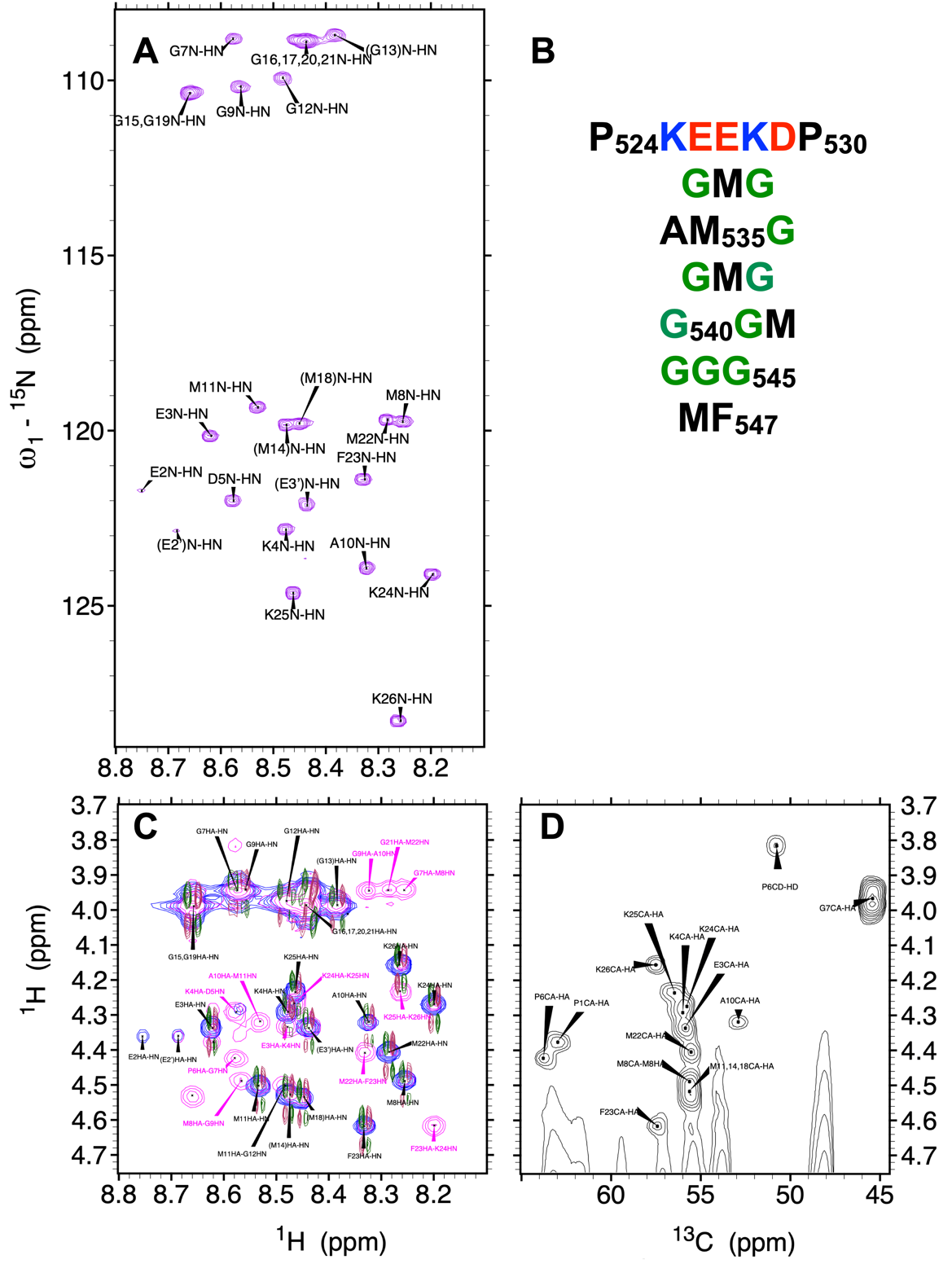


**A**. ^1^H-^15^N HSQC spectrum of the C-terminal tail of mHsp60. Ambiguous assignments are shown in parentheses.

**B**. The residues of the mHsp60 C-terminal tail, which are invisible to CryoEM or X-ray diffraction. Cationic, anionic, nonpolar and glycine residues are colored blue, red, black and green, respectively.

**C**. ^1^HN-^1^Hα region of 2D ^1^H-^1^H COSY (maroon/dark green), TOCSY (blue) and NOESY (magenta) spectra. Inter-residue crosspeaks are colored maroon and intra-residues crosspeaks are colored black.

**D**. ^1^Hα/^13^Cα region of 2D ^1^H-^13^C HSQC spectrum.

**Supporting Figure 5**: NMR Parameters and CD Spectra of GroEL and mHsp60 C-terminal Segment (complete)


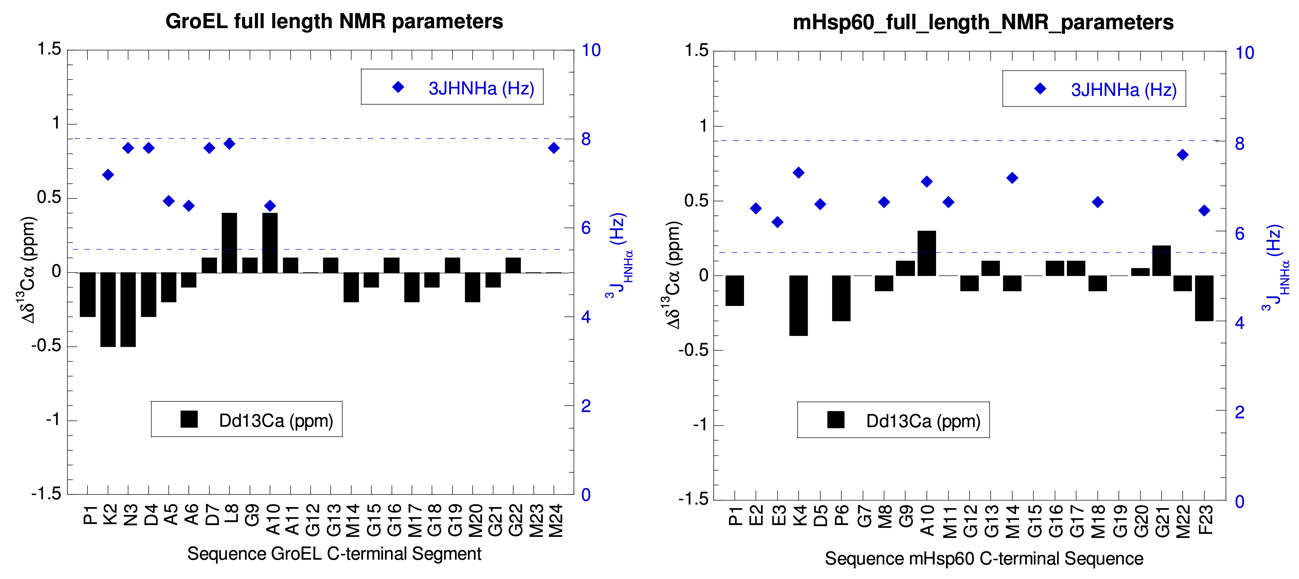


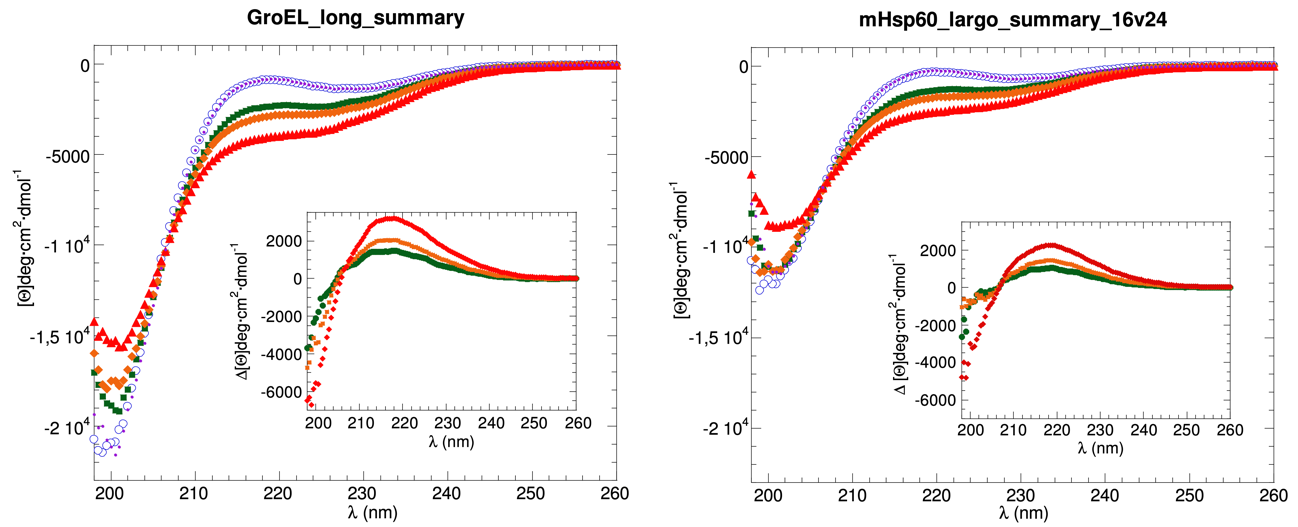


^13^Cα conformational chemistry shifts (black bars, left y-axis) and ^3^J_HNHα_ coupling constants (blue diamonds, right y-axis) for the GroEL (top left panel) and mHsp60 (top right panel) complete C-terminal segments. Blue dashed horizontal lines mark the range of constants (between 5.5 & 8.0 Hz) expected for statistical coil or polyproline II conformations.

Far UV-CD spectra recorded at 5ºC (blue), 25ºC (green) 37ºC (orange) and 65ºC (red) and after recooling to 5ºC (purple) for the C-terminal tails of GroEL (bottom left panel) and mHsp60 (bottom right panel). Insets show the difference CD spectra for 5ºC-25ºC (green), 5ºC-37ºC (orange) and 5ºC-65ºC (red).

**Supporting Figure 6:** Second one μs MD simulation of GroEL_7_·GroES_7_·ADP_7_ with Amber99SB-disp force field


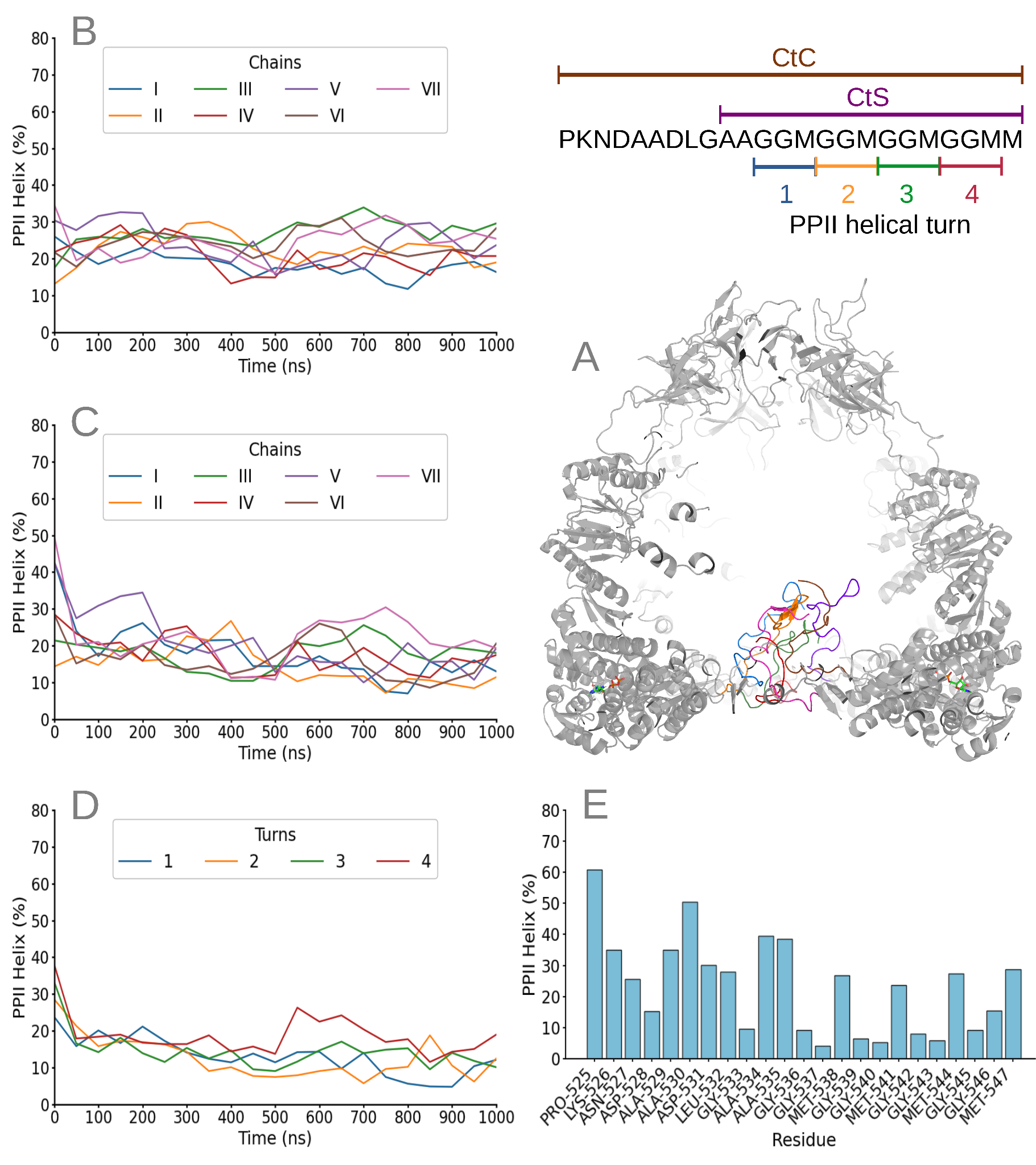


Schematics (top right) show the sequence of the complete (CtC) and short (CtS) C-terminal sequences and the PPII helix numbering.

**A.**  Frame after one μs of simulation showing GroEL_7_·GroES_7_ folded domains in gray, ADP in green, blue, red and orange for C, N, O and P atoms, respectively, and the seven C-terminal segments in blue, orange, green, red, purple, brown and pink.

**B** & **C**. PPII helical content averaged over 50 ns for each of the seven C-terminal complete (**B**) or short (**C**) segments.

**D**. PPII helical content averaged over 50 ns in each of the four GGM repeats, colored blue, yellow, green and red for the first, second, third and fourth repeat, respectively.

**E**. Mean per-residue PPII helical population averaged over the whole one μs simulation run.

Results from two other independent one μs simulations are shown in **Figure 3** and **Supp. Figure 6**.

**Supporting Figure 7:** Third one μs MD simulation of GroEL_7_·GroES_7_·ADP_7_ with Amber99SB-disp force field


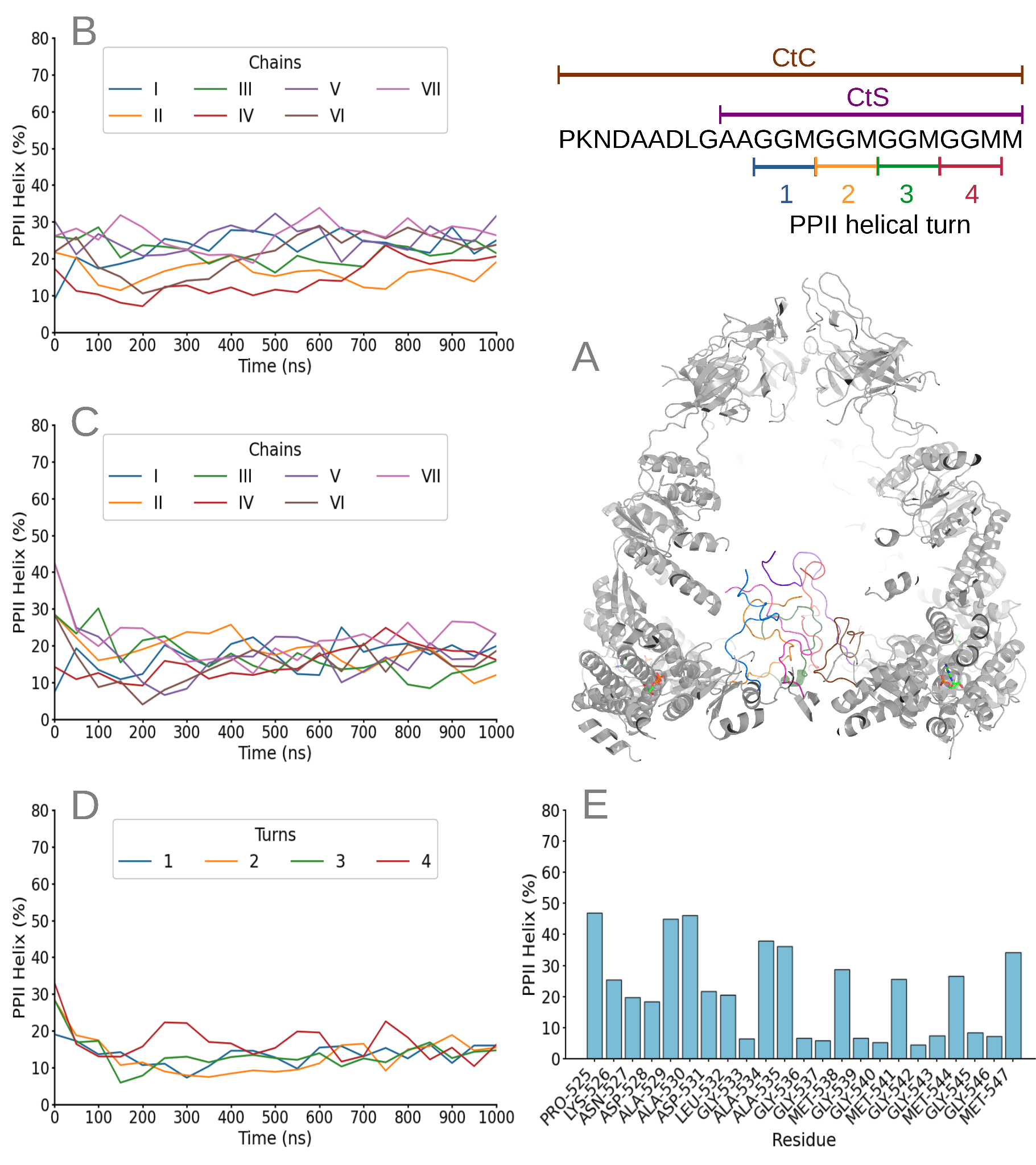


Schematics (top right) show the sequence of the complete (CtC) and short (CtS) C-terminal sequences and the PPII helix numbering.

**A.** Frame after one μs of simulation showing GroEL_7_·GroES_7_ folded domains in gray, ADP in green, blue, red and orange for C, N, O and P atoms, respectively, and the seven C-terminal segments in blue, orange, green, red, purple, brown and pink.

**B** & **C**. PPII helical content averaged over 50 ns for each of the seven C-terminal complete (**B**) or short (**C**) segments.

**D**. PPII helical content averaged over 50 ns in each of the four GGM repeats, colored blue, yellow, green and red for the first, second, third and fourth repeat, respectively.

**E**. Mean per-residue PPII helical population averaged over the whole one μs simulation run.

Results from two other independent one μs simulations are shown in **Figure 3** and **Supp. Figure 5**.

**Supporting Figure 8:** Second one μs MD simulation of GroEL_7_·GroES_7_·ADP_7_ with CHARMM36m force field


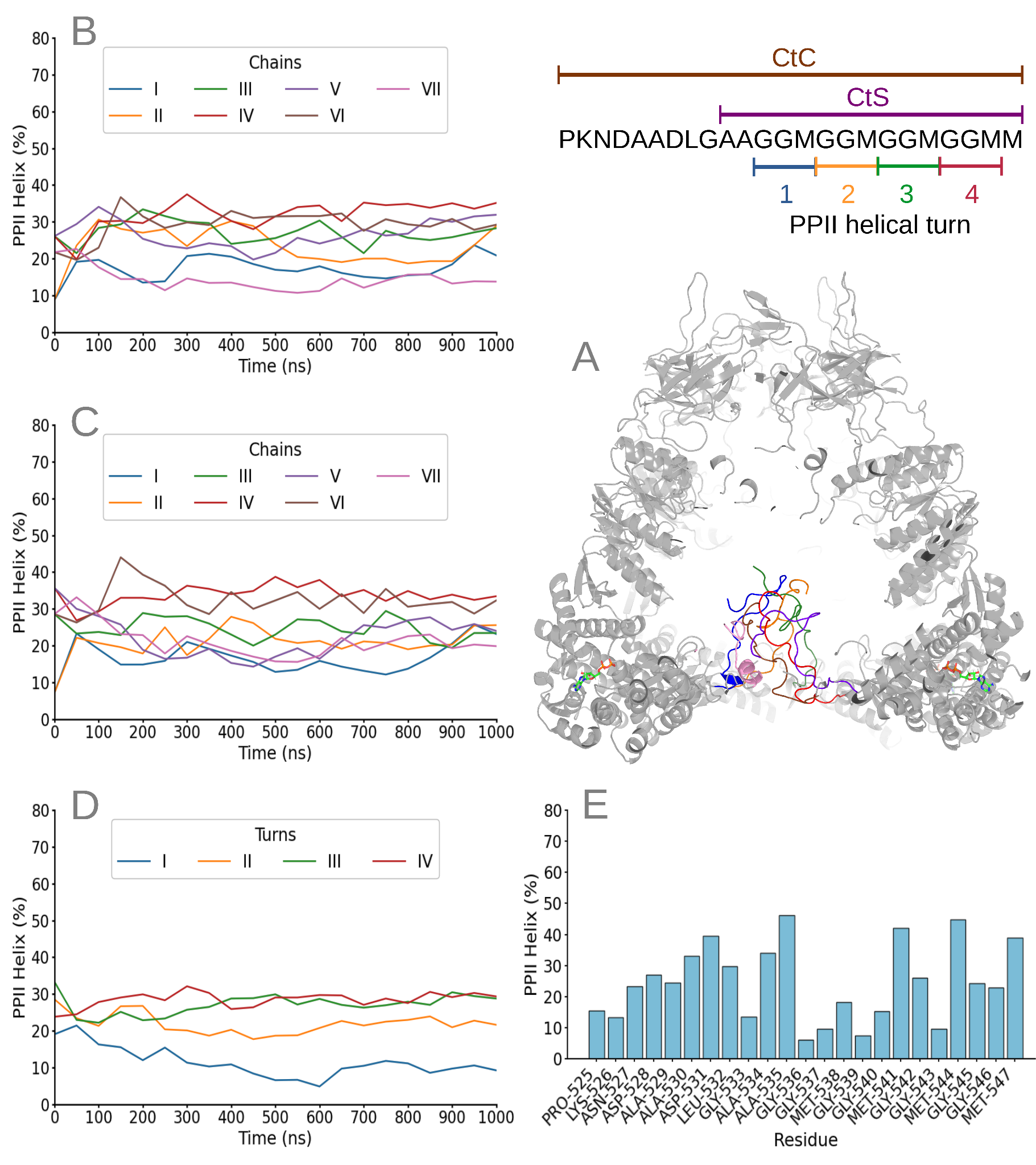


Schematics (top right) show the sequence of the complete (CtC) and short (CtS) C-terminal sequences and the PPII helix numbering.

**A.** Frame after one μs of simulation showing GroEL_7_·GroES_7_ folded domains in gray, ADP in green, blue, red and orange for C, N, O and P atoms, respectively, and the seven C-terminal segments in blue, orange, green, red, purple, brown and pink.

**B** & **C**. PPII helical content averaged over 50 ns for each of the seven C-terminal complete (**B**) or short (**C**) segments.

**D**. PPII helical content averaged over 50 ns in each of the four GGM repeats, colored blue, yellow, green and red for the first, second, third and fourth repeat, respectively.

**E**. Mean per-residue PPII helical population averaged over the whole one μs simulation run.

Results from two other independent one μs simulations are shown in **Figure 4** and **Supp. Figure 9**.

**Supporting Figure 9:** Third one μs MD simulation of GroEL_7_·GroES_7_·ADP_7_ with CHARMM36m force field


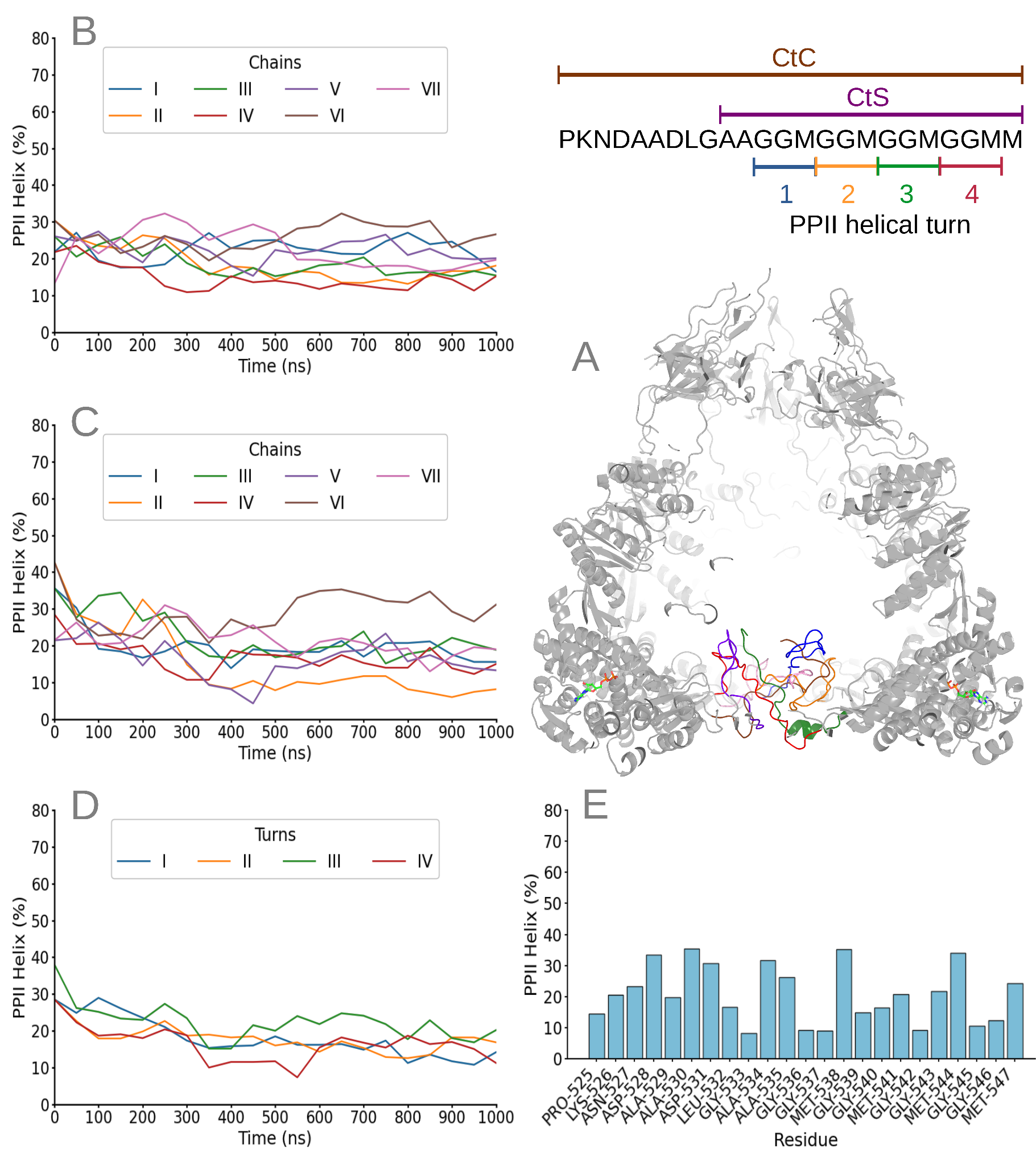


Schematics (top right) show the sequence of the complete (CtC) and short (CtS) C-terminal sequences and the PPII helix numbering.

**A.** Frame after one μs of simulation showing GroEL_7_·GroES_7_ folded domains in gray, ADP in green, blue, red and orange for C, N, O and P atoms, respectively, and the seven C-terminal segments in blue, orange, green, red, purple, brown and pink.

**B** & **C**. PPII helical content averaged over 50 ns for each of the seven C-terminal complete (**B**) or short (**C**) segments.

**D**. PPII helical content averaged over 50 ns in each of the four GGM repeats, colored blue, yellow, green and red for the first, second, third and fourth repeat, respectively.

**E**. Mean per-residue PPII helical population averaged over the whole one μs simulation run.

Results from two other independent one μs simulations are shown in **Figure 4** and **Supp. Figure 8**.

**Supporting Figure 10**: Second one μs MD simulation of GroEL_7_·GroES_7_·ATP_7_ or GroEL_7_·GroES_7_·ATP_7_ plus HP35 with Amber99SB-disp force field


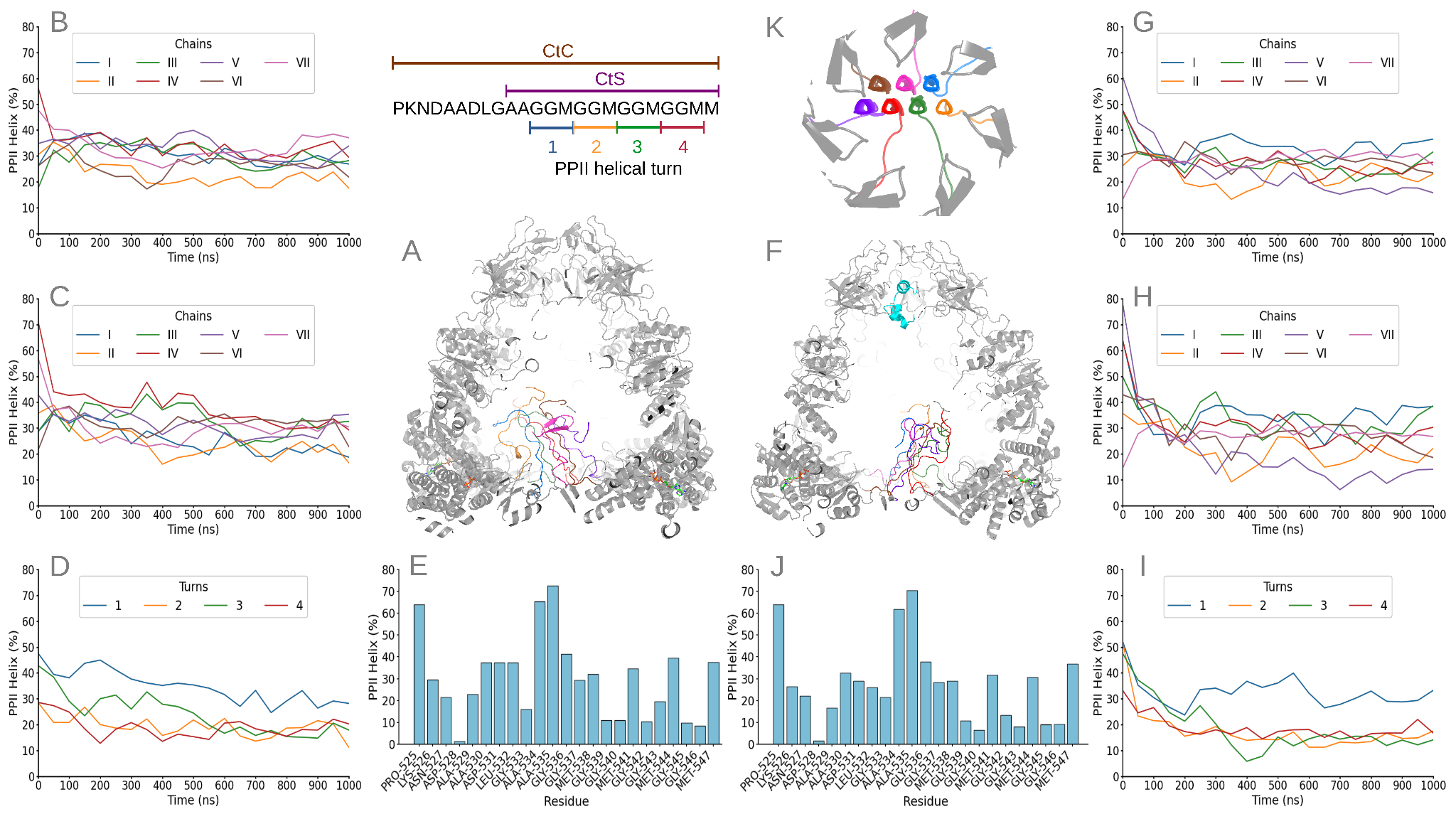


Schematic diagrams in the top central part of the figure show the sequence of the complete (CtC) and short (CtS) C-terminal segments, the numbering of the PPII helical turns and a top-down view of the GGM repeats of the seven segments arranged in a PPII helical bundle.

**GroEL_7_·GroES_7_ with 7ATP:**

**A**. Frame after one μs of simulation of GroEL_7_·GroES_7_ with 7ATP without HP35 (panels **A-D**) showing GroEL_7_·GroES_7_ folded domains in gray, ATP in green, blue, red and orange for C, N, O and P atoms, respectively. The C-terminal segments are colored blue (I), orange (II), green (III), red (IV), purple (V), brown (VI) and pink (VII).

**B** & **C**. PPII helical content averaged over 50 ns for each of the seven C-terminal complete (**B**) or short (**C**) segments.

**D**. PPII helical content averaged over 50 ns in each for the four GGM repeats, colored blue, yellow, green and red for the first, second, third and fourth repeat, respectively.

**E**. Mean per-residue PPII helical population averaged over the whole one μs simulation run.

**GroEL_7_·GroES_7_ with 7ATP + HP35:**

**F**. Frame after one μs of simulation with HP35 (in cyan) and GroEL_7_·GroES_7_ folded domains in gray, ATP in green, blue, red and orange for C, N, O and P atoms, respectively. The C-terminal segments are colored blue (I), orange (II), green (III), red (IV), purple (V), brown (VI) and pink (VII).

**G** & **H**. PPII helical content averaged over 50 ns for each of the seven C-terminal complete (**G**) or short (**H**) segments.

**I**. PPII helical content averaged over 50 ns in each GGM repeat for the four GGM repeats, colored blue, yellow, green and red, for the first, second, third and fourth repeat, respectively.

**J**. Mean per-residue PPII helical population averaged over the whole one μs simulation run.

Results from two other independent one μs MD simulations are shown in **Figure 5** and **Supp. Figure 11**.

**Supporting Figure 11**: Third one μs MD simulation of GroEL_7_·GroES_7_·ATP_7_ or GroEL_7_·GroES_7_·ATP_7_ plus HP35 with Amber99SB-disp force field


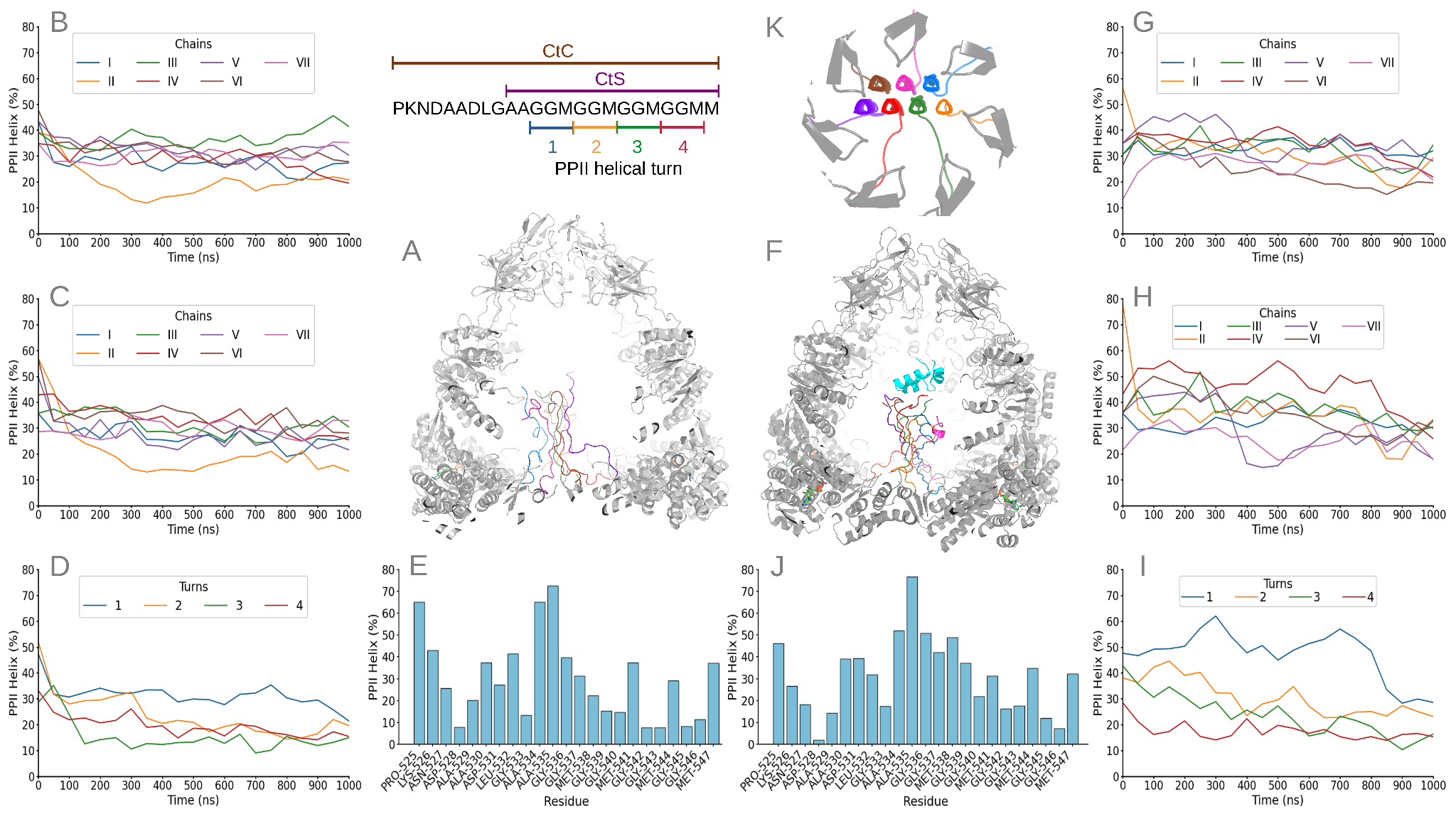


Schematic diagrams in the top central part of the figure show the sequence of the complete (CtC) and short (CtS) C-terminal segments, the numbering of the PPII helical turns and a top-down view of the GGM repeats of the seven segments arranged in a PPII helical bundle.

**GroEL_7_·GroES_7_ with 7ATP:**

**A**. Frame after one μs of simulation of GroEL_7_·GroES_7_ with 7ATP without HP35 (panels **A-D**) showing GroEL_7_·GroES_7_ folded domains in gray, ATP in green, blue, red and orange for C, N, O and P atoms, respectively. The C-terminal segments are colored blue (I), orange (II), green (III), red (IV), purple (V), brown (VI) and pink (VII).

**B** & **C**. PPII helical content averaged over 50 ns for each of the seven C-terminal complete (**B**) or short (**C**) segments.

**D**. PPII helical content averaged over 50 ns in each for the four GGM repeats, colored blue, yellow, green and red for the first, second, third and fourth repeat, respectively.

**E**. Mean per-residue PPII helical population averaged over the whole one μs simulation run.

**GroEL_7_·GroES_7_ with 7ATP + HP35:**

**F**. Frame after one μs of simulation with HP35 (in cyan) and GroEL_7_·GroES_7_ folded domains in gray, ATP in green, blue, red and orange for C, N, O and P atoms, respectively. The C-terminal segments are colored blue (I), orange (II), green (III), red (IV), purple (V), brown (VI) and pink (VII).

**G** & **H**. PPII helical content averaged over 50 ns for each of the seven C-terminal complete (**G**) or short (**H**) segments.

**I**. PPII helical content averaged over 50 ns in each GGM repeat for the four GGM repeats, colored blue, yellow, green and red, for the first, second, third and fourth repeat, respectively.

**J**. Mean per-residue PPII helical population averaged over the whole one μs simulation run.

Results from two other independent one μs MD simulations are shown in **Figure 5** and **Supp. Figure 10**.

**Supporting Figure 12**: Second one μs MD simulation of GroEL_7_·GroES_7_·ATP_7_ or GroEL_7_·GroES_7_·ATP_7_ plus HP35 with CHARMM36m force field


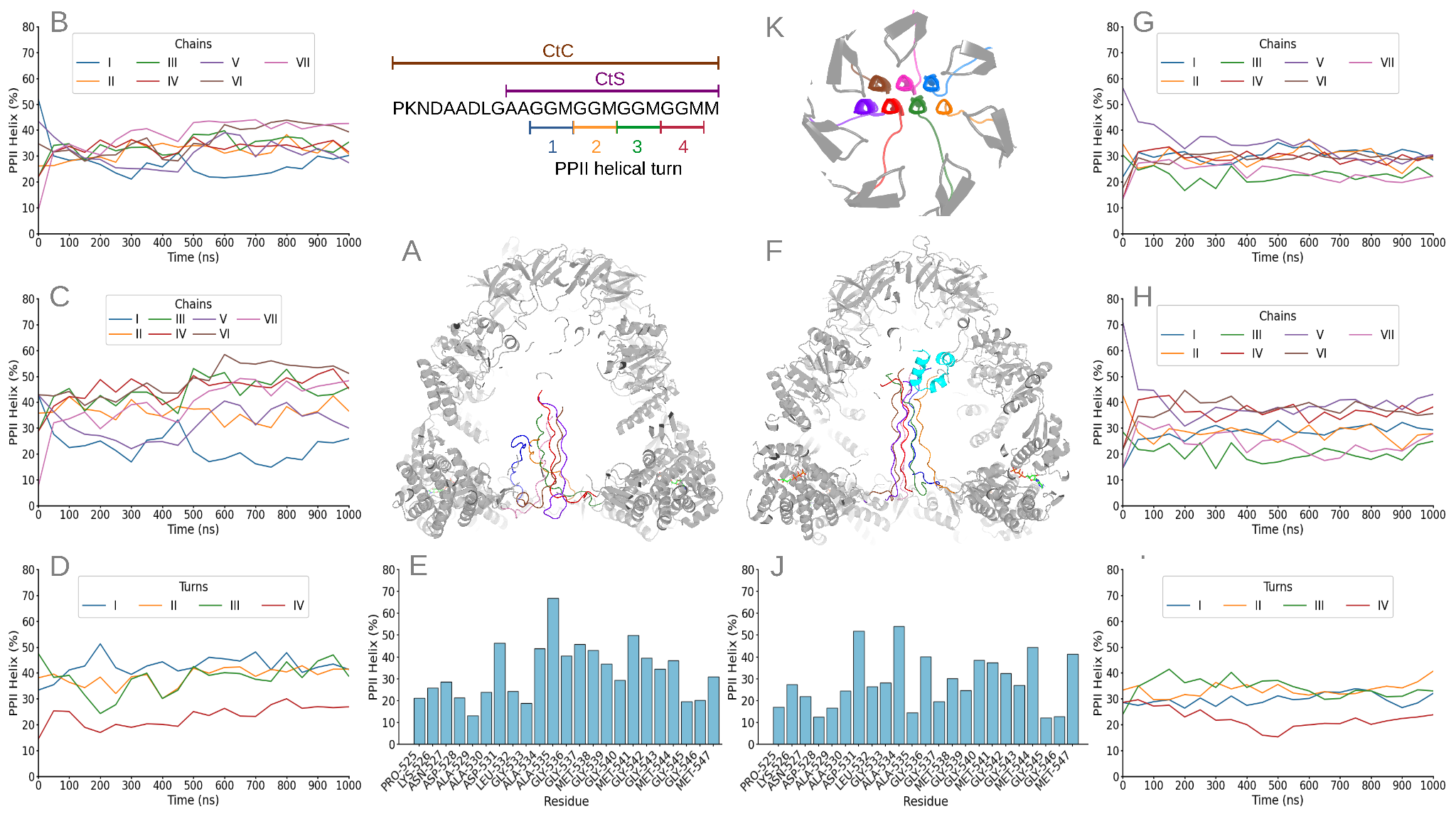


Schematic diagrams in the top central part of the figure show the sequence of the complete (CtC) and short (CtS) C-terminal segments, the numbering of the PPII helical turns and a top-down view of the GGM repeats of the seven segments arranged in a PPII helical bundle.

**GroEL_7_·GroES_7_ with 7ATP:**

**A**. Frame after one μs of simulation of GroEL_7_·GroES_7_ with 7ATP without HP35 (panels **A-D**) showing GroEL_7_·GroES_7_ folded domains in gray, ATP in green, blue, red and orange for C, N, O and P atoms, respectively. The C-terminal segments are colored blue (I), orange (II), green (III), red (IV), purple (V), brown (VI) and pink (VII).

**B** & **C**. PPII helical content averaged over 50 ns for each of the seven C-terminal complete (**B**) or short (**C**) segments.

**D**. PPII helical content averaged over 50 ns in each for the four GGM repeats, colored blue, yellow, green and red for the first, second, third and fourth repeat, respectively.

**E**. Mean per-residue PPII helical population averaged over the whole one μs simulation run.

**GroEL_7_·GroES_7_ with 7ATP + HP35:**

**F**. Frame after one μs of simulation with HP35 (in cyan) and GroEL_7_·GroES_7_ folded domains in gray, ATP in green, blue, red and orange for C, N, O and P atoms, respectively. The C-terminal segments are colored blue (I), orange (II), green (III), red (IV), purple (V), brown (VI) and pink (VII).

**G** & **H**. PPII helical content averaged over 50 ns for each of the seven C-terminal complete (**G**) or short (**H**) segments.

**I**. PPII helical content averaged over 50 ns in each GGM repeat for the four GGM repeats, colored blue, yellow, green and red, for the first, second, third and fourth repeat, respectively.

**J**. Mean per-residue PPII helical population averaged over the whole one μs simulation run.

Results from two other independent one μs MD simulations are shown in **Figure 6** and **Supp. Figure 13**.

**Supporting Figure 13**: Third one μs MD simulation of GroEL_7_·GroES_7_·ATP_7_ or GroEL_7_·GroES_7_·ATP_7_ plus HP35 with CHARMM36m force field


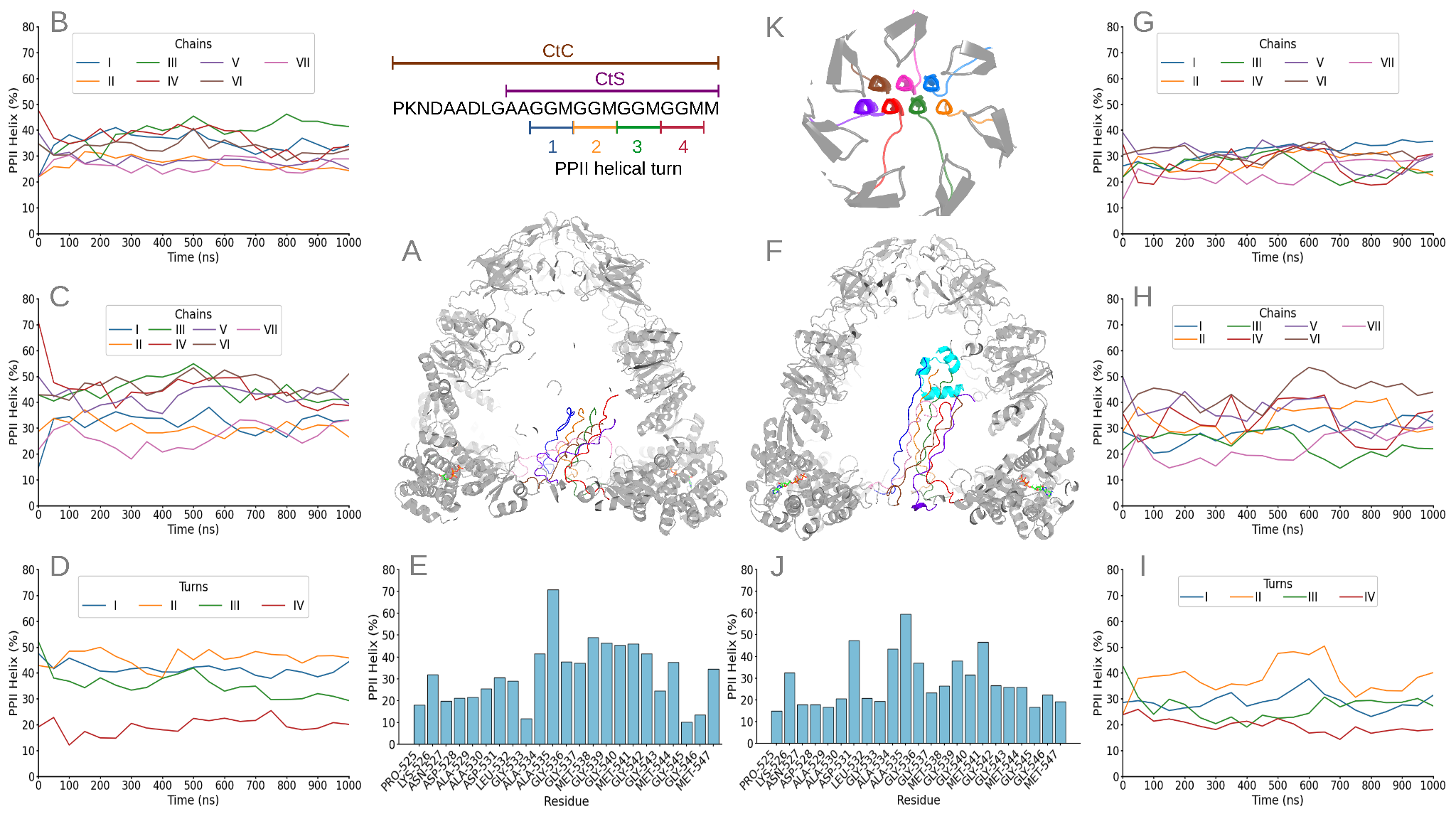


Schematic diagrams in the top central part of the figure show the sequence of the complete (CtC) and short (CtS) C-terminal segments, the numbering of the PPII helical turns and a top-down view of the GGM repeats of the seven segments arranged in a PPII helical bundle.

**GroEL_7_·GroES_7_ with 7ATP:**

**A**. Frame after one μs of simulation of GroEL_7_·GroES_7_ with 7ATP without HP35 (panels **A-D**) showing GroEL_7_·GroES_7_ folded domains in gray, ATP in green, blue, red and orange for C, N, O and P atoms, respectively. The C-terminal segments are colored blue (I), orange (II), green (III), red (IV), purple (V), brown (VI) and pink (VII).

**B** & **C**. PPII helical content averaged over 50 ns for each of the seven C-terminal complete (**B**) or short (**C**) segments.

**D**. PPII helical content averaged over 50 ns in each for the four GGM repeats, colored blue, yellow, green and red for the first, second, third and fourth repeat, respectively.

**E**. Mean per-residue PPII helical population averaged over the whole one μs simulation run.

**GroEL_7_·GroES_7_ with 7ATP + HP35:**

**F**. Frame after one μs of simulation with HP35 (in cyan) and GroEL_7_·GroES_7_ folded domains in gray, ATP in green, blue, red and orange for C, N, O and P atoms, respectively. The C-terminal segments are colored blue (I), orange (II), green (III), red (IV), purple (V), brown (VI) and pink (VII).

**G** & **H**. PPII helical content averaged over 50 ns for each of the seven C-terminal complete (**G**) or short (**H**) segments.

**I**. PPII helical content averaged over 50 ns in each GGM repeat for the four GGM repeats, colored blue, yellow, green and red, for the first, second, third and fourth repeat, respectively.

**J**. Mean per-residue PPII helical population averaged over the whole one μs simulation run.

Results from two other independent one μs MD simulations are shown in **Figure 6** and **Supp. Figure 12**.

**Supporting Figure 14:** First one μs MD simulation of GroEL_7_·GroES_7_·ADP_7_ with pre-assembled PPII helical bilayer and CHARMM36m force field


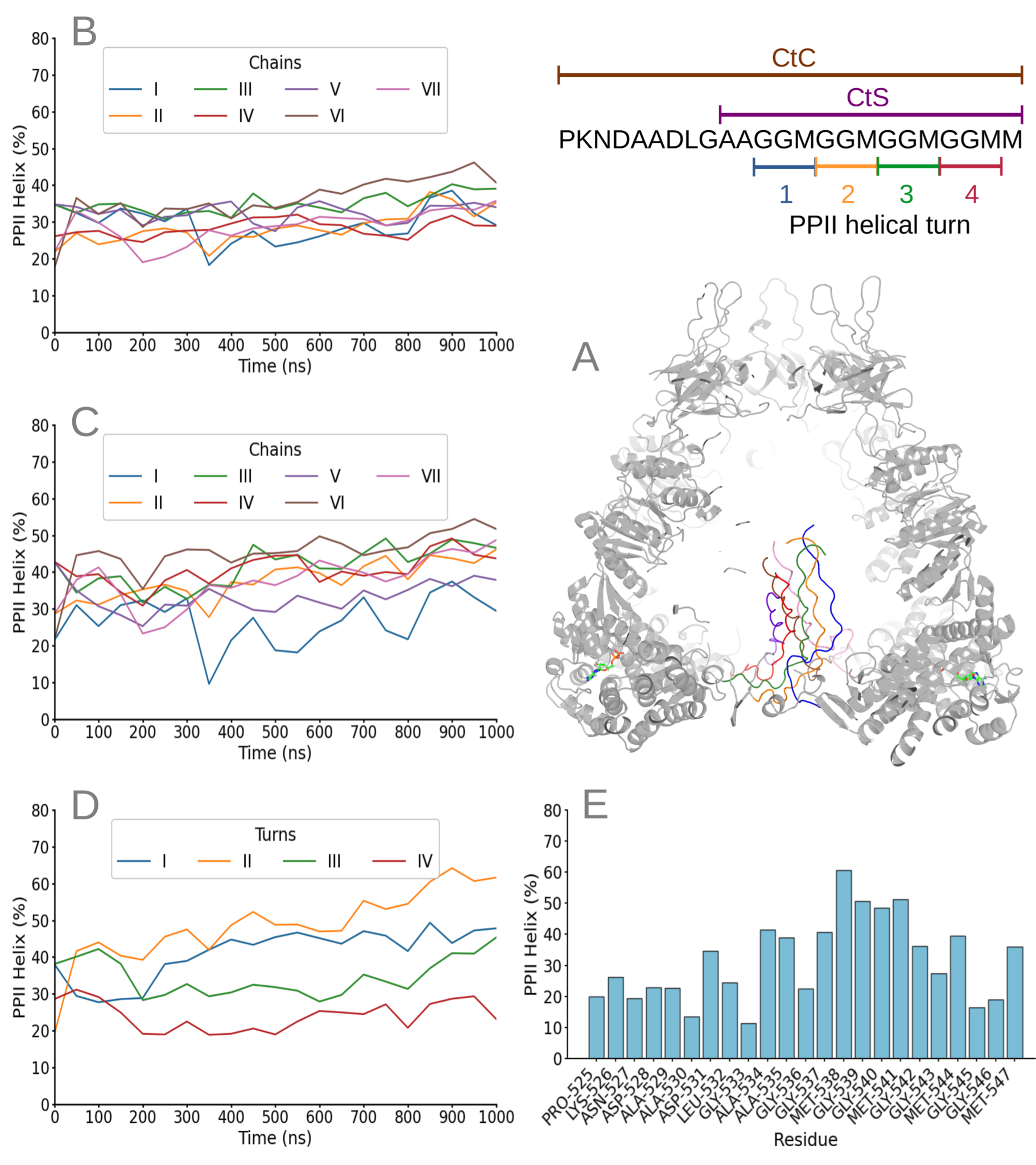


Schematics (top right) show the sequence of the complete (CtC) and short (CtS) C-terminal sequences and the PPII helix numbering.

**A.** Frame after one μs of simulation showing GroEL_7_·GroES_7_ folded domains in gray, ADP in green, blue, red and orange for C, N, O and P atoms, respectively, and the seven C-terminal segments in blue, orange, green, red, purple, brown and pink.

**B** & **C**. PPII helical content averaged over 50 ns for each of the seven C-terminal complete (**B**) or short (**C**) segments.

**D**. PPII helical content averaged over 50 ns in each of the four GGM repeats, colored blue, yellow, green and red for the first, second, third and fourth repeat, respectively.

**E**. Mean per-residue PPII helical population averaged over the whole one μs simulation run.

Results from two other independent one μs simulations are shown in **Supp. Figure 15** and **Supp. Figure 16**.

**Supporting Figure 15:** Second one μs MD simulation of GroEL_7_·GroES_7_·ADP_7_ with pre-assembled PPII helical bilayer and CHARMM36m force field


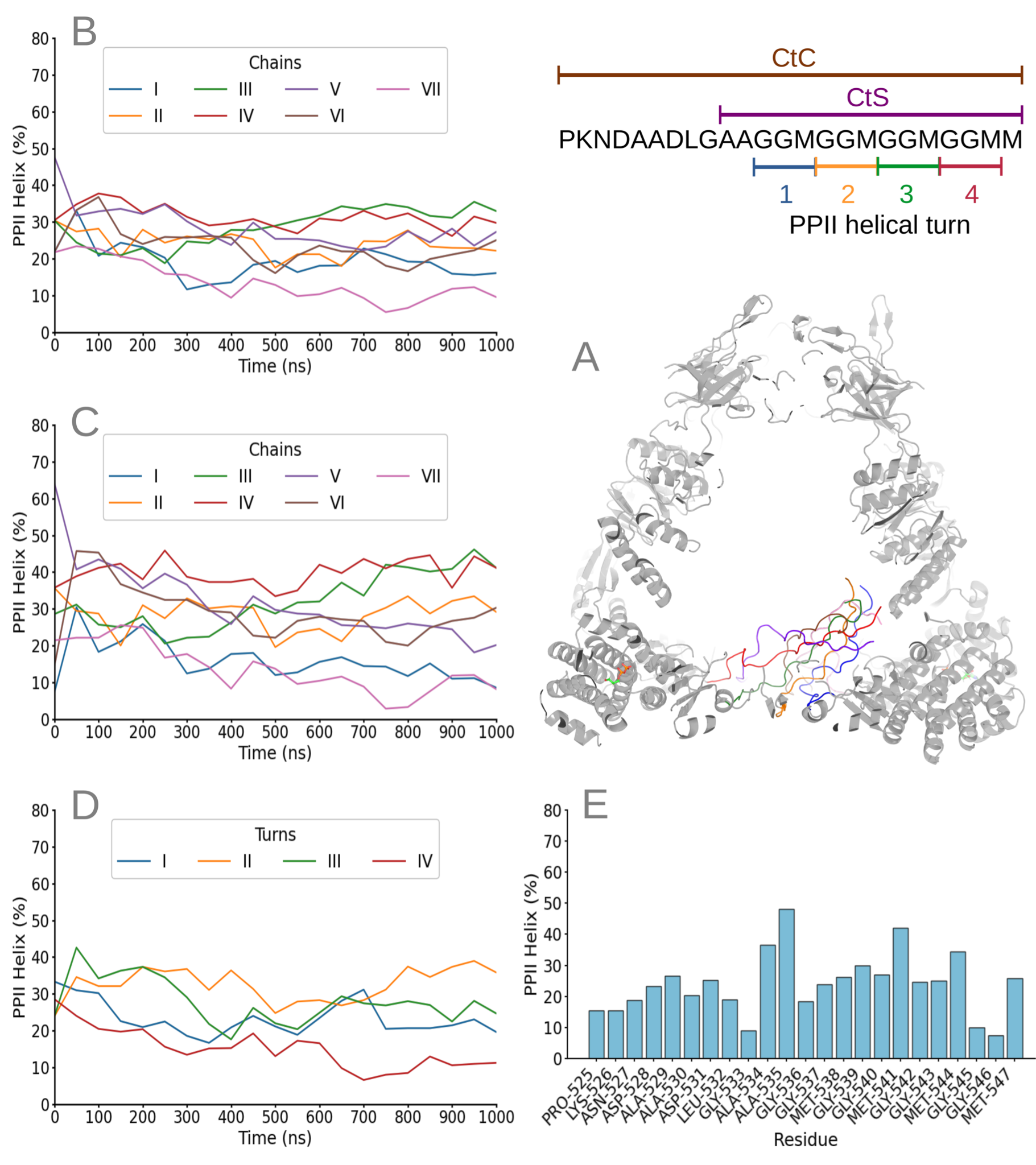


Schematics (top right) show the sequence of the complete (CtC) and short (CtS) C-terminal sequences and the PPII helix numbering.

**A.** Frame after one μs of simulation showing GroEL_7_·GroES_7_ folded domains in gray, ADP in green, blue, red and orange for C, N, O and P atoms, respectively, and the seven C-terminal segments in blue, orange, green, red, purple, brown and pink.

**B** & **C**. PPII helical content averaged over 50 ns for each of the seven C-terminal complete (**B**) or short (**C**) segments.

**D**. PPII helical content averaged over 50 ns in each of the four GGM repeats, colored blue, yellow, green and red for the first, second, third and fourth repeat, respectively.

**E**. Mean per-residue PPII helical population averaged over the whole one μs simulation run.

Results from two other independent one μs simulations are shown in **Supp. Figure 14** and **Supp. Figure 16**.

**Supporting Figure 16:** Third one μs MD simulation of GroEL_7_·GroES_7_·ADP_7_ with pre-assembled PPII helical bilayer and CHARMM36m force field


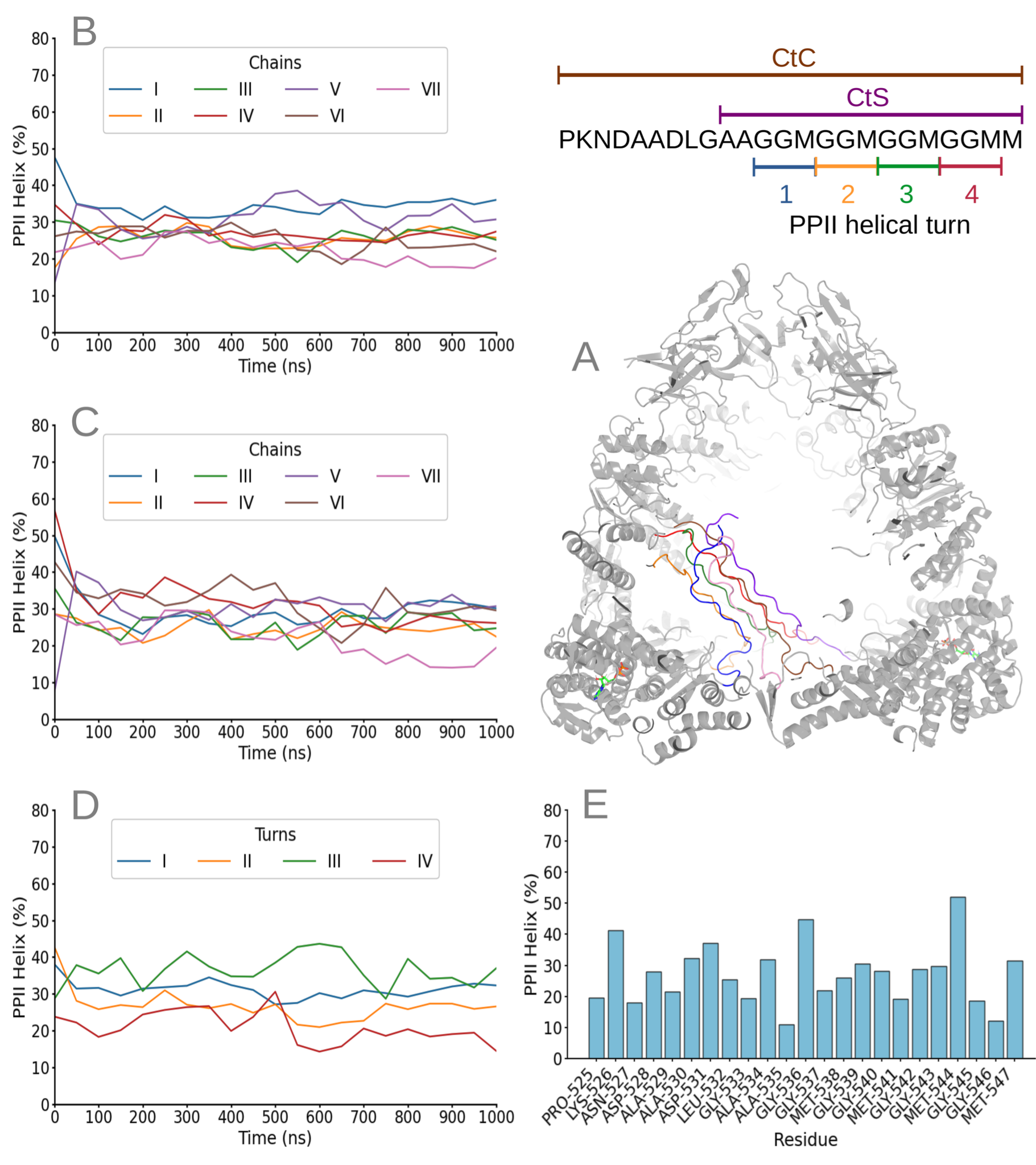


Schematics (top right) show the sequence of the complete (CtC) and short (CtS) C-terminal sequences and the PPII helix numbering.

**A.** Frame after one μs of simulation showing GroEL_7_·GroES_7_ folded domains in gray, ADP in green, blue, red and orange for C, N, O and P atoms, respectively, and the seven C-terminal segments in blue, orange, green, red, purple, brown and pink.

**B** & **C**. PPII helical content averaged over 50 ns for each of the seven C-terminal complete (**B**) or short (**C**) segments.

**D**. PPII helical content averaged over 50 ns in each of the four GGM repeats, colored blue, yellow, green and red for the first, second, third and fourth repeat, respectively.

**E**. Mean per-residue PPII helical population averaged over the whole one μs simulation run.

Results from two other independent one μs simulations are shown in **Supp.** **Figures 14** and **15**

**Supporting Figure 17**:

NMR Spectra of *Arabadopsis thaliana* and wheat Cpn60α C-terminal segments


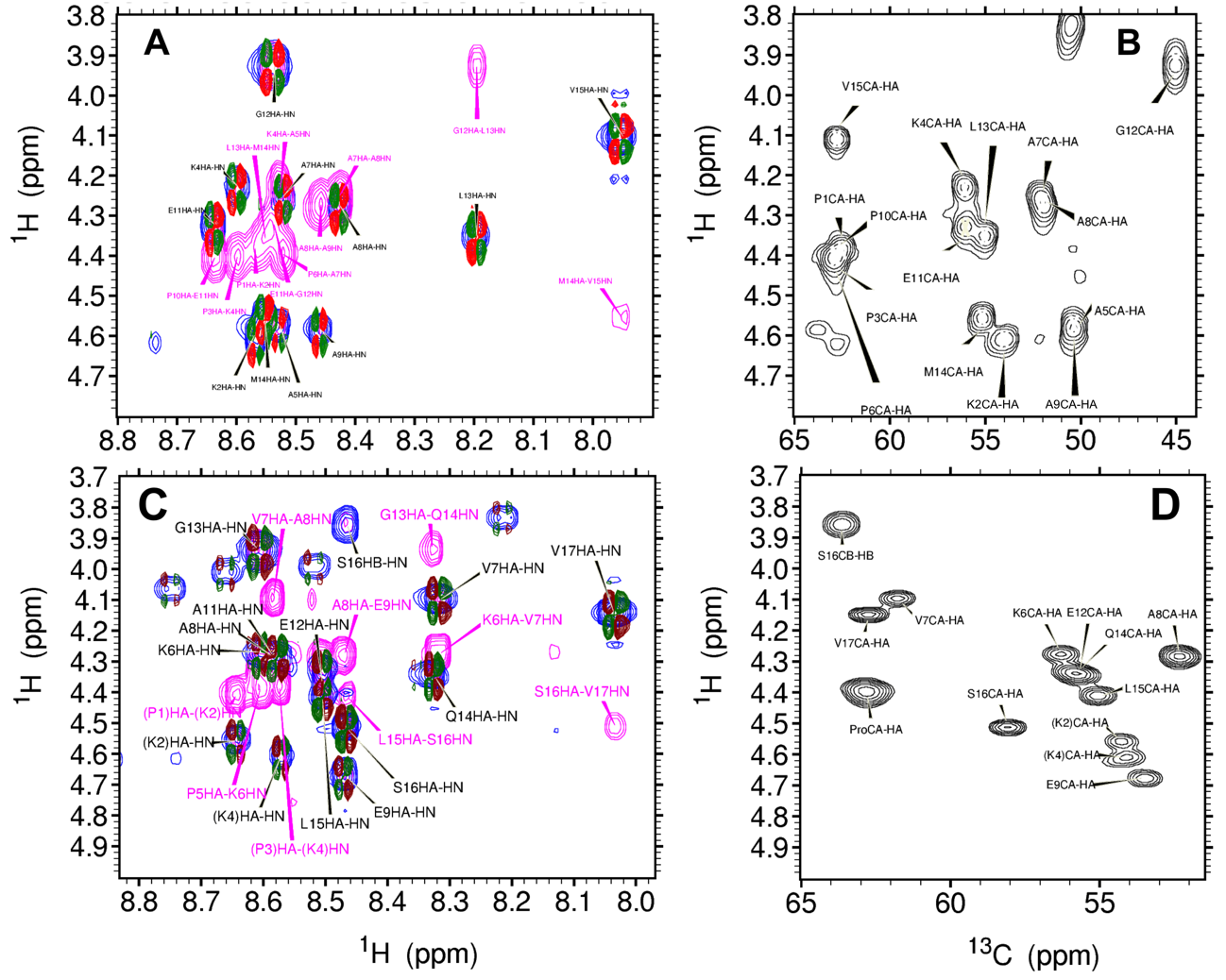


**A.** ^1^HN-^1^Hα region of 2D ^1^H-^1^H TOCSY (blue), NOESY (magenta), and COSY (maroon/dark green) spectra of *A. thaliana* Cpn60α Ct. Intraresidue crosspeaks are labeled in black and sequential NOESY crosspeaks are labeled in magenta.

**B**. ^1^Hα-^13^Cα region of the 2D ^1^H-^13^C HSQC spectrum of *A. thaliana* Cpn60α Ct. The signals arising from the proline residues are overlapped. Contours are plotted with a 1.4 multiplication factor.

**C**. ^1^HN-^1^Hα region of 2D ^1^H-^1^H TOCSY (blue), NOESY (magenta), and COSY (maroon/dark green) spectra of Wheat Cpn60α Ct. Intraresidue crosspeaks are labeled in black and sequential NOESY crosspeaks are labeled in magenta. Assignments for K2 and K4 as well as P1 and P3 (in parentheses) are ambiguous.

**D**. ^1^Hα-^13^Cα region of the 2D ^1^H-^13^C HSQC spectrum of Wheat Cpn60α Ct. Assignments

for K2 and K4 (in parenthesis) are ambiguous and the signals arising from the proline

residues (labeled “Pro”) are overlapped. Contours are plotted with a 1.4 multiplication

factor.

**Supporting Figure 18**

NMR Parameters and CD Spectra of *A. thaliana* and wheat Cpn60β


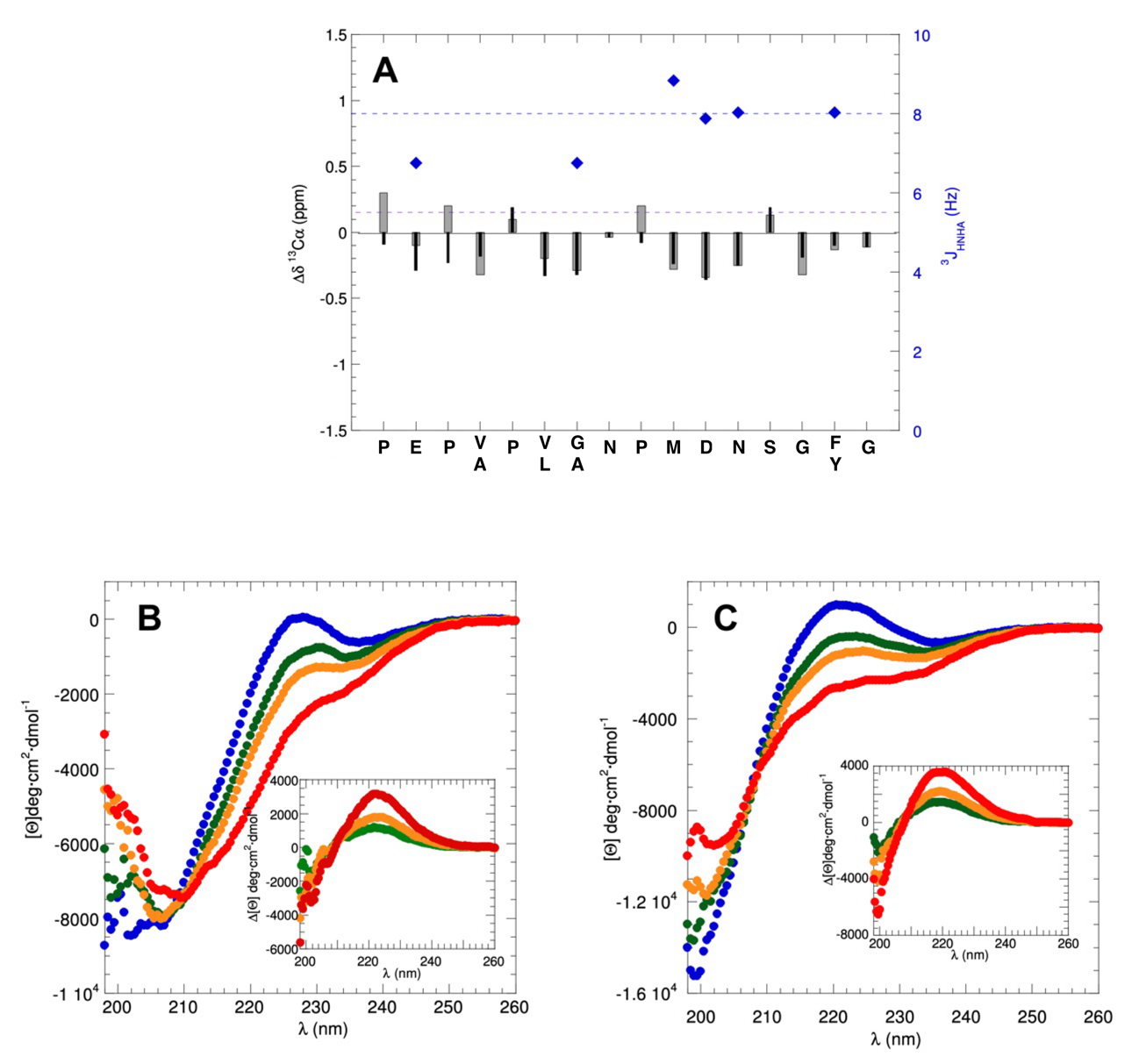


**A.** ^13^Cα conformational chemistry shifts of *A. thaliana* (black narrow bars, left y-axis, top sequence) and wheat (gray wide bars, left y-axis, bottom sequence) and wheat ^3^J_HNHα_ coupling constants (blue diamonds, right y-axis) Cpn60β C-terminal segments. Blue dashed horizontal lines mark the range of constants (between 5.5 & 8.0 Hz) expected for statistical coil or PPII conformations.

Far UV-CD spectra recorded at 5ºC (blue), 25ºC (green) 37ºC (orange) and 65ºC (red) and after recooling to 5ºC (purple) for the C-terminal tails of *A. thaliana* Cpn60β (panel **B**) and wheat Cpn60β (panel **C**). Insets show the difference CD spectra for 5ºC-25ºC (green), 5ºC-37ºC (orange) and 5ºC-65ºC (red).

**XXII**. Link for Supporting Videos 1-18 of MD simulations.

Please clic this link to a Zenodo record for the Supporting Videos of the MS simulations, whose file sizes exceeded the journal server limit.

<https://zenodo.org/records/15784832?token=eyJhbGciOiJIUzUxMiJ9.eyJpZCI6IjI5ZDBiMjg1LTAwZWMtNDY1Yi1iOTRjLWI3MTJiNTFkMWExYSIsImRhdGEiOnt9LCJyYW5kb20iOiJmNjU4MTJjZTU3ZDQ5YTg0Zjg4YWYxNWU5YWJkN2EwYiJ9.2FaTyLjIqRNpVaUNFOhB2cSS5e_oRecwYmti74Bsv9CEGPvczW1MqDhko761CfB-uiBdjaz19g02eEAXXrOm3Q>
