# Supplementary material for: The C‐terminal tails of GroEL and its mitochondrial and chloroplastic homologs adopt polyproline II helices
Source: Protein Sci. 2025 Oct 29;34(11):e70354. doi: 10.1002/pro.70354 (PMC12571646; doi:10.1002/pro.70354)
Supplement: Supplementary file 2 — Data S2: Supporting Information [file PRO-34-e70354-s002.docx]

**Supporting Movie Text**

Supplementary Videos were prepared from the molecular dynamics simulations of GroEL_7_·GroES_7_ with 7 ADP or 7 ATP bound, different initial conformations of the C-termini and using distinct force fields as detailed in the following table.

| **Supplementary Movies** | **Proteins** | **Ligands** | **Initial state of the C-termini** | **Force Field** |
| --- | --- | --- | --- | --- |
| 1, 2 & 3 | GroEL_7_·GroES_7_ | 7 ADP | disordered | Amber99SB-disp |
| 4, 5 & 6 | GroEL_7_·GroES_7_ | 7 ADP | disordered | CHARMM36m |
| 7, 8 & 9 | GroEL_7_·GroES_7_ | 7 ATP | Bundle of 7 PPII helices | Amber99SB-disp |
| 10, 11 & 12 | GroEL_7_·GroES_7_ | 7 ATP | Bundle of 7 PPII helices | CHARMM36m |
| 13, 14 & 15 | GroEL_7_·GroES_7_ + HP35 client protein | 7 ATP | Bundle of 7 PPII helices | Amber99SB-disp |
| 16, 17 & 18 | GroEL_7_·GroES_7_ + HP35 client protein | 7 ATP | Bundle of 7 PPII helices | CHARMM36m |

Link for Supporting Videos 1-18 of MD simulations.

Please clic this link to a Zenodo record for the Supporting Videos of the MS simulations, whose file sizes exceeded the journal server limit.

<https://zenodo.org/records/15784832?token=eyJhbGciOiJIUzUxMiJ9.eyJpZCI6IjI5ZDBiMjg1LTAwZWMtNDY1Yi1iOTRjLWI3MTJiNTFkMWExYSIsImRhdGEiOnt9LCJyYW5kb20iOiJmNjU4MTJjZTU3ZDQ5YTg0Zjg4YWYxNWU5YWJkN2EwYiJ9.2FaTyLjIqRNpVaUNFOhB2cSS5e_oRecwYmti74Bsv9CEGPvczW1MqDhko761CfB-uiBdjaz19g02eEAXXrOm3Q>
